# Intraspecific Variation in Protists: Clues for Microevolution from *Poteriospumella lacustris* (Chrysophyceae)

**DOI:** 10.1093/gbe/evz171

**Published:** 2019-08-06

**Authors:** Stephan Majda, Jens Boenigk, Daniela Beisser

**Affiliations:** Department of Biodiversity, Duisburg-Essen, Germany

**Keywords:** chrysomonad flagellates, genome comparison, whole genome sequencing, ploidy

## Abstract

Species delimitation in protists is still a challenge, attributable to the fact that protists are small, difficult to observe and many taxa are poor in morphological characters, whereas most current phylogenetic approaches only use few marker genes to measure genetic diversity. To address this problem, we assess genome-level divergence and microevolution in strains of the protist *Poteriospumella lacustris*, one of the first free-living, nonmodel organisms to study genome-wide intraspecific variation.

*Poteriospumella lacustris* is a freshwater protist belonging to the Chrysophyceae with an assumed worldwide distribution. We examined three strains from different geographic regions (New Zealand, China, and Austria) by sequencing their genomes with the Illumina and PacBio platforms.

The assembled genomes were small with 49–55 Mb but gene-rich with 16,000–19,000 genes, of which ∼8,000 genes could be assigned to functional categories. At least 68% of these genes were shared by all three species. Genetic variation occurred predominantly in genes presumably involved in ecological niche adaptation. Most surprisingly, we detected differences in genome ploidy between the strains (diploidy, triploidy, and tetraploidy).

In analyzing intraspecific variation, several mechanisms of diversification were identified including SNPs, change of ploidy and genome size reduction.

## Introduction

Genetic variation permits flexibility and survival of a population under changing environmental conditions (Reed and Frankham 2003) and leads over time to genetic differences between strains or populations (diversification) from different geographic regions or environments. Genetic variation therefore provides insights into the evolutionary history of a species (Knoll 1994; Darling et al. 2004).

Eukaryotes can have large intraspecific variation in DNA content (Parfrey et al. 2008). For example, recent studies of intraspecific genetic variation using DNA fingerprinting techniques in aquatic phytoplankton have identified high levels of diversity. Average gene diversity, which gives the probability that two alleles chosen at random from a population will be different from each other, range from 39% to 88% (Godhe and Rynearson 2017). Logares et al. (2009) found among five dinoflagellates a genetic diversity between 20% and 90%. Several further studies demonstrated a surprisingly high intraspecific genetic diversity in both marine and limnic species (John et al. 2004; Evans et al. 2005; Rynearson and Armbrust 2005; Hayhome et al. 2007). However, these experiments were all based on microsatellites or DNA fingerprinting and reflect only a small part of the entire intraspecific variation in microeukaryotes.

A high intraspecific variation may not be considered surprising as under a neutral model, a population’s genetic diversity depends (besides the gene’s mutation rate) on the effective population size (Kimura 1979). Because of their short generation time and huge population size (Watts et al. 2013), the genetic variation in protists may thus be enormous. Despite a potentially high intraspecific variation, the mechanisms of microevolution and with that of speciation may not necessarily correspond to those discussed for multicellular organisms: Many protist taxa are sexually reproducing (Raikov 1995; Heywood and Magee 1976) but a separation of gene pools between subpopulations does not necessarily occur (Fenchel and Finlay 2004) as protists have a high potential for long-range and persistent dispersal and many taxa show a cosmopolitan distribution (e.g., Finlay 2002).

On the other hand, protist populations, in particular in freshwater lakes, are separated as lakes are considered to act like islands (as in the theory of island biogeography; MacArthur and Wilson 1967); that is, despite the huge population size and the ease of dispersal, populations may be (at least temporarily) separated. Corresponding to these considerations, limited distribution and geographic separation have been shown to apply also to protist populations (Fernandez et al. 2017; Bestová et al. 2018; Boenigk et al. 2018). However, the geographic differences on the community level, in particular the low contribution of geography as demonstrated for protists, do not necessarily indicate evolutionary separation of subpopulations but may result from regional extinctions of species or temporal fluctuations between plankton and seed bank. Despite recent indications for geographic isolation of protist communities, it remains uncertain to what extent this separation applies to the level of subpopulation, that is, to intraspecific variation, which would provide a basis for speciation by geographic separation. The huge population sizes of protists and the relative ease of dispersal may, hinder or even prevent speciation by geographic separation as known for many multicellular organisms.

The extent of intraspecific genomic variation in protists is still obscure. Here, we examine the genomic molecular variation between three clonal strains of the heterotrophic chrysophyte species *Poteriospumella lacustris*. The strains were collected in different geographical regions (JBC07 in China, JBM10 in Austria, and JBNZ41 in New Zealand; Boenigk et al. 2005). Despite the geographic remote sampling sites they have identical SSU and ITS sequences except for one base deviating in the ITS region of strain JBNZ41 (from 835 bp) and show similar ecophysiological characteristics (Boenigk et al. 2005, 2007). Despite this high similarity backing up the affiliation with the same species, transcriptome studies indicated a high intraspecific variation (Beisser et al. 2017). It remains unclear to what extent the reported gene content variation is artificial due to differential gene expression or rather reflects true genomic intraspecific variation. Further, the transcriptome studies suggested a different degree of genetic variation for different pathways but again conclusions remained vague due to incomplete gene coverage.

In order to resolve the puzzle of genetic variation within this free-living protist species we here study the intraspecific genome-level variation. We test the hypothesis that genes coding for primary and secondary pathways differently reflect the accumulation of intraspecific molecular variation. In particular, we expect genes affiliated with the basal metabolism to be conserved, whereas genes affiliated with the secondary metabolism and with pathways directly interacting with the environment should be more diverse due to adaptations to changing environmental conditions; that is, the gene variation in primary metabolism is assumed to be higher than in secondary metabolism. We further test whether intraspecific variation with respect to the accumulation of point mutations, changes of ploidy and genome reduction is weak as would be expected based on the close relatedness. Or alternatively, whether the variation is high as would be expected due to the large population sizes and the global distribution of this species. We further analyze intraspecific genomic variation for indications of evolutionary differentiation and recent population bottlenecks.

In order to address the above hypotheses we sequenced the three strains using Illumina and PacBio sequencing platforms and the assembled genomes were examined by comparing, for example, gene content, gene density, SNPs, proportion of repetitive regions, ploidy, and GC content. We further identified gene functions and pathways using the *Kyoto Encyclopedia of Genes and Genomes* (KEGG) database (Kanehisa and Goto 2000).

## Materials and Methods

### Cultivation and Sequencing

Three clonal strains of *P.lacustris* (JBM10, JBNZ41, JBC07; Findenig et al. 2010) from the culture collection of the working group were cultivated according to Hahn et al. (2003) in NSY medium under a light:dark cycle of 12:12 h at 16 C. Before harvesting, the axenic cultures were tested for contaminations using light microscopy. The DNA was isolated (Bio-Budget, my-Budget DNA Mini Kit, Krefeld, Germany) and sequenced by sequencing provider (BGI, Hong Kong) using the Illumina Hiseq XTen technology (insert size 300 bp, BGI in-house reagents) for the strains JBC07, JBM10, and JBNZ41 and PacBio RSII for strain JBM10. PacBio processing was done with gTUBE (Covaris, USA) for shearing genome DNA to 20 kb, DNA Template Prep Kit 3.0, DNA/Polymerase Binding Kit and DNA sequencing Reagent 4.0 (Pacific Biosciences, USA).

### Genome Assembly

Unless otherwise stated, the default settings were used for the following programs. A Snakemake (Koster and Rahmann 2012) automated workflow was created to process the sequencing data. We benchmarked the N50 statistic of different assemblers (SPAdes, v3.10.1 [Nurk et al. 2013], Celera, v8.3 [Myers et al. 2000], ABySS, v2.0.2 [Simpson et al. 2009], CANU, v1.5 [Koren et al. 2017]) and chose supported by literature, for example Forouzan et al. (2017), Sovic et al. (2016) and the implementation of hybrid approaches, the following assembler and procedure: First CANU (with parameters: genomeSize = 96m correctedErrorRate = 0.105; Koren et al. 2017) was used to assemble the PacBio reads of strain JBM10. The genome size parameter was chosen based on estimates from Olefeld et al. (2018). Subsequently, with SPAdes (v3.10.1, with parameters: –untrustedcontigs; Nurk et al. 2013) the Illumina reads of each strain were assembled using the PacBio reads of JBM10 as scaffolding template. By this hybrid assembly approach a simplified de Bruijn graph was constructed and overlaid with an assembly graph of long contigs at graph edges to close gaps and resolve repeats (Antipov et al. 2016) combining low error rates with long scaffolds. The Illumina reads were decisive for the assembled sequence allowing comparisons between strain sequences later on. After assembly, contigs smaller than 500 bp were discarded. About 23,000 18S DNA sequences of Chromista (from NCBI) were blasted (BlastN, v2.5.0, with parameters: -percidentity 99; Boratyn et al. 2012) against the scaffolds to validate the correctness of the strains and to exclude the possibility of contamination. Genome size was first estimated by kmers (*k* = 21) with KMC (Dlugosz et al. 2017) and GenomeScope (Underwood et al. 2017). However, we changed the method due to discrepancies (see supplementary table S1, Supplementary Material online) with the estimation from nuclear staining and flow cytometry (Olefeld et al. 2018). Hence, the length of all contigs was summed up for each strain. Genome characteristics were compared with all available stramenopile genomes from NCBI (last accessed February 2019). Genomes smaller than 50 Mb were excluded, since these are usually parasites or organelles, which are not suitable for comparison.

The Benchmarking Universal Single-Copy Orthologs (BUSCO) software (v3.0.2; Simao et al. 2015) was used to verify the existence of all essential orthologous genes and to measure genome completeness. For BUSCO data sets of protists and eukaryota were used. During data submission to NCBI, the genomes were aligned to publicly available organelle genomes and classified mitochondrial contigs.

### Gene Prediction

Different approaches of gene prediction were tested (details see supplementary file, Supplementary Material online). In the final approach, the gene pattern of *Arabidopsis thaliana* was chosen as model for gene prediction with AUGUSTUS (v3.3 with parameters: –species = arabidopsis –gff3 = on –singlestrand = true –UTR = off; Stanke et al. 2006). Mapping the RNA sequences of the strains (Beisser et al. 2017) to the predicted genes with Bowtie (v.2.2.8 with parameters: –very-sensitive-local; Langmead et al. 2009) functioned as validation of the prediction procedure.

Mitochondrial genes were predicted and annotated by aligning the genes of the *Ochromonas danica* mitochondrial genome (from NCBI, ACCESSION number: NC_002571) with the contigs, that were identified as mitochondrial sequences, using Minimap2 (2.16-r922; with parameters: -c -G 80 K; Li 2018).

### Gene Clustering

**Fig. 1.**
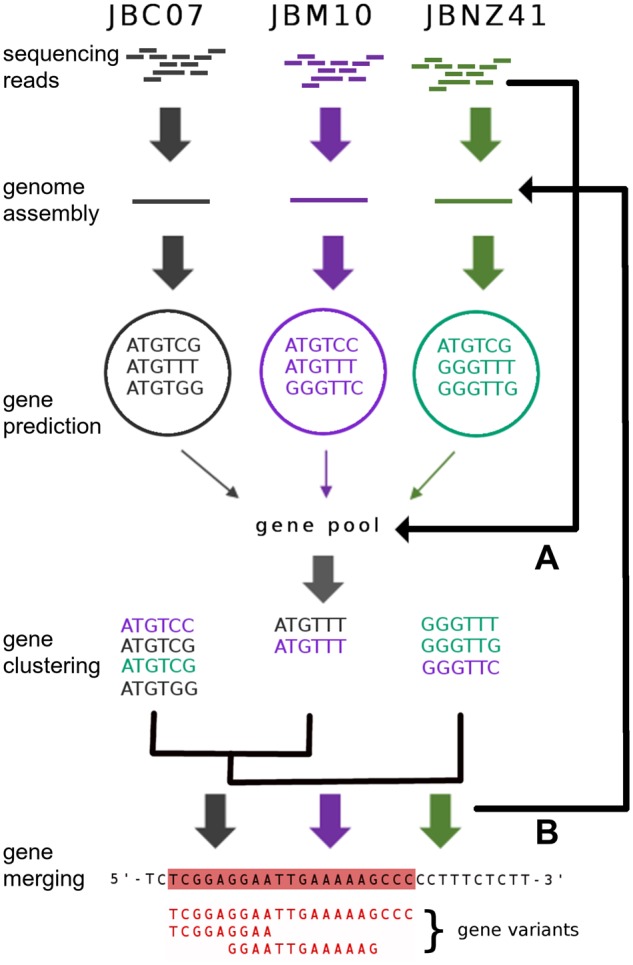
—Flow chart of gene prediction and analysis. AUGUSTUS (Stanke et al. 2006) predicted genes based on assemblies of each strain genome. Predicted genes were pooled and clustered with CD-HIT (Li and Godzik 2006). (*A*) Sequenced reads of each strain were mapped against the gene pool. (*B*) Clustered genes were aligned against each genome. Genes overlapping on the same strand were merged. This procedure prevents overlooking of genes and combines gene duplicates and variants. Step *A*: All reads of each strain could be map back. Step *B*: Genes aligned either specific or to multiple genomes.

Since the genome assembly yielded many contigs, the gene prediction could likely miss several genes between or at the edge of the contigs. Mapping reads from one strain against exclusive genes from another strain showed which genes could possibly be build with the read set. We merged the predicted genes of all strains and clustered them with CD-HIT (v4.6.8 with parameters: cd-hit-est -n 8 -M 20000 -T 18 -s 0.8 -aL 0.8 -aS 0.8 -G 0; Li and Godzik 2006). Gene clusters were merged by their consensus sequence. In order to prevent overlooking of genes, the reads of each strain were mapped with BWA (Li and Durbin 2009) against this pool of predicted genes. Including this information, all genes could be covered completely (coverage > 5×) by the sequencing reads of each strain (see fig. 1). To exclude that genes were missing due to incorrect assemblies, the strain specific genes of JBC07 and JBNZ41 were further aligned to the PacBio-sequenced contigs of JBM10. We aligned the pool of predicted genes against each genome to detect correct genes with Minimap2 (v.2.9-r720; with parameters: -x splice -G 80K). The alignment of predicted genes of JBM10 against its genome was used as a validation of whether spliced genes were correctly aligned. Multiple genes aligning on the same strand with an overlap of at least 10% of the gene length were declared as one gene or a splice-variant of the gene. Genes aligning on several genomes were declared as shared genes whereas genes aligning to only one genome were specific.

### Gene Annotation

Using Diamond (v0.9.10.111; Buchfink et al. 2015), we aligned the predicted genes to the KEGG (Release 2014-06-23; Kanehisa and Goto 2000) and UniProt database (Release 2017-09-18; Pundir et al. 2017) to obtain KEGG Orthology (KO) identifiers. Both databases complemented each other. In case of inconsistencies the lower *e*-value defined the assignment. KO identifiers operate as unique flags for a functionally orthologous group of genes. Thereby, a species independent gene annotation and comparison is made possible. Furthermore, the “KEGG Mapper—Reconstruct Module” tool was used to reveal the metabolic pathways. Thereby, a module describes a pathway that is necessary for a defined function. A functional module consisting of only two genes was confirmed if it was completely, whereas modules with more than two genes were allowed to miss a maximum of one gene. Inspection of the essential primary metabolic pathways (relevant for energy production, metabolism of carbohydrates, lipids, amino acid and genetic information processing) was used as completeness check for the genome.

We searched for signal peptides in all predicted genes of each strain with SignalP (v5.0; with parameters: -batch 100000, Almagro Armenteros et al. 2019) and HECTAR (Gschloessl et al. 2008) to identify proteins that are translocated across membranes. 25% of the genes with the lowest validation score identified by SignalP were filtered out to increase specificity. These genes were grouped in the category *genes encoding organelle targeted proteins* and analyzed like the other KEGG functional groups.

### Genome Similarity, Ploidy Estimation, and Analysis of Repetitive Regions

Genome similarity based on the average nucleotide identity (ANI) was calculated with FastAni (v1.1 Jain et al. 2017) for the whole genome. We aligned the mitochondrial sequences pairwise between the strains with Minimap2 (v2.16-r922; with parameters: -cx asm5 -Y) to determine similarity between them. Since they only differ in a few bases, the mitochondrial sequences were additionally mapped (Minimap2 with parameters: -c –splice) against the PacBio contigs of JBM10 to exclude all mitochondrial sequences originate from the same PacBio template.

RepeatScout (v1.0.5; Price et al. 2005) was used to construct a de novo repetitive sequences library for each strain. Including this libraries RepeatMasker (v4.0.7; http://www.repeatmasker.org; last accessed August 2019) identified repetitive sequences and masked them. Genome ploidy was estimated by nQuire (version from April 5, 2018; with parameters: nQuire create -c 20 -q 15 with following denoise step; Weiß et al. 2018). Additionally, ploidy was determined for the repeat masked genomes as well as separately for each contig longer than 10,000 bp. For the contig-wise ploidy estimation, it was evaluated whether the probability for a certain ploidy level was at least 10% higher compared with the others. In this case ploidy counts were normalized by contig length. The tool nQuire uses different frequencies of SNP mutations on heterozygous alleles to determine the ploidy. Additionally, ploidy was estimated based on *k*-mer frequencies with *smudgeplot* (v0.1.3 with parameters: -k21 -m300 -ci1 -cs10000; https://github.com/tbenavi1/smudgeplot; last accessed August 2019). This package determines the number of *k*-mer pairs differing by one SNP and compares them to their relative coverage.

### Gene Analysis

GATK haplotypecaller (v4.0.6.0 with parameters: ploidy 4 –emit-ref-confidence GVCF –output-mode EMIT ALL CONFIDENT SITES –annotate-with-num-discovered-alleles true –max-reads-per-alignment-start 0; McKenna et al. 2010) was used for variant calling on the whole genome. Here, all strains were consistently evaluated with parameter *-ploidy 4*, which served as upper boundary, but had no influence on the lower ploidy. Variants were excluded if read depth was <8 or the variant occurred in <10% of the reads. Next, the genes with their variants were extracted and subdivided by KEGG functional categories. For each kind of variation (SNP, insertion, deletion) the occurrence per gene was determined. Additionally, we compared the gene variation pairwise for each strain. If at any position there was a mismatch between two genes and no allelic variation could induce a match we listed a mutation. To normalize the data, the number of mutations were divided by the length of the gene to obtain the mutation rate of the allele. Additionally, identical genes between strains were counted. Afterwards a one-way ANOVA test verified significant differences (*P*-value < 0.01) within a functional group for each allelic variation (SNP, insertion, deletion) between the three strains. A following Wilcoxon signed-rank tested which of the functional groups differed significantly. For the mutation analysis between strains, we repeated these steps, expect Kruskal–Wallis test was performed instead of ANOVA, because the data were not normally distributed.

Genes were divided into shared (if they could be aligned by Minimap2 (v.2.9-r720; with parameters: -x splice -G 80 K to more than one genome)) or exclusive genes and counted. The R package eulerr (v4.1.0; Larsson 2018) was used to visualize the amount of shared genes. The gene density (*d*) was calculated for the number of genes (*n*) by the formula:

(1) $$d=\frac{n}{\sum {\mathrm{bp}}_{\mathrm{contigs}>500bp}}*10^{6}$$

Gene densities were compared with 197 protists from the Ensembl Protists database (accessed December 2017, https://protists.ensembl.org).

## Results

### Genome Assembly and Gene Prediction

Illumina sequencing generated 80.8 (JBC07), 79.6 (JBM10), and 109 (JBNZ41) million 150 bp-long paired end reads and 207,000 PacBio reads (JBM10) with a total length of 1,505 Mbps (reads lengths: mean = 7,243 bp, median = 7,130 bp). The sequence data were assembled into draft genomes for each strain (see table 1). The assembly of the PacBio reads for JBM10 produced 695 contigs (N50 = 143,709 bp). The combination of short Illumina reads and the assembly of PacBio reads as template resulted in a high number of contigs (9,122–13,826, see table 2). The obtained contigs had a coverage between 116× and 153×. The proportion of repeat regions amounted to 12–16%. The mitochondrial genome of each strain could be assembled almost completely (JBC07: 38,850 bp, 2 contigs; JBM10: 38,860 bp, 2 contigs; JBNZ41: 38,814 bp, 1 contig). About 760 genes with signal peptides were identified. Mapping against the PacBio contigs of JBM10 showed all mitochondrial sequences were based on the strain specific Illumina reads. From the BUSCO reference gene set for eukaryotes 81.5–83.8% genes were recovered. However, in the gene set for protists 54.5–55.8% genes were recovered (see supplementary table S2, Supplementary Material online). Taking into account the genome reduction in *P. lacustris* and analyzing the annotated genes using KEGG, verified that essential metabolic pathways were covered (see supplementary table S14, Supplementary Material online).

**Table 1:** Genome Size Estimations

*P. lacustris* strain	JBC07	JBM10	JBNZ41
Total genome size	157.2	96.5	177.5
(Flow cytometry) [Mb]			
Haploid genome size	52.4	48.3	44.4
(Flow cytometry) [Mb]			
(Haploid) assembly size [Mb]	49.4	54.7	52.8
Estimated ploidy level	Triploid	Diploid	Tetraploid

Note.—The genome size was estimated in Olefeld et al. (2018) by nuclear staining and flow cytometry. The haploid genome size of the flow cytometry was recalculated by the total size and the ploidy estimation. Assembly size reflects the sum over the length of all contigs longer than 500 bp.

**Table 2:** Overview of Sequencing and Genome Characteristics

Species	JBC07	JBM10	JBNZ41	*Ectocarpus siliculosus*	*Nannochloropsis oceanica*	average stramenopiles
# contigs	9,122	9,400	13,826	13,533	32	—
N50	40,792	52,370	24,662	32,613	—	—
Coverage	116	128	153	—	—	—
GC %	53.1	53.1	52.9	53.5	54.1	49.7
Repetitive regions %	13.2	16.6	12.4	—	—	—
Predicted genes	55,941	60,147	62,248	—	7k–10k	—
Final genes	17,315	16,915	19,494	16,269	7k–10k	8,368
Annotated genes	7,453	7,960	8,525	—	222	—
Gene length (median)	1,612	1,887	1,331	243	455	—
Gene length (mean)	3,169	3,582	2,871	226	687	—
Gene density [Mb^–1^]	350	309	369	83	—	269
Ratio coding DNA to total %	55.5	55.3	53.0	13.2	—	—
Average nucleotide identity (ANI) [%]	97.5	98.0	97.4	—	—	—

Note.—Predicted genes were aligned to the genome. Overlapping genes were merged to final genes. The gene density describes the number of genes per MB, with the gene lengths varying widely, whereas the ratio of coding DNA to total DNA is independent of the gene length. The genome similarity was expressed by the ANI. The stramenopiles data are based on the average of the statistics data of all available stramenopiles genomes (*n* = 97) from NCBI (https://www.ncbi.nlm.nih.gov/genome/? term=stramenopiles; last accessed August 2019). *Ectocarpus siliculosus* data also originate from NCBI. *Nannochloropsis oceanica* data are based on NCBI and Wang et al. (2014).

**Fig. 2.**
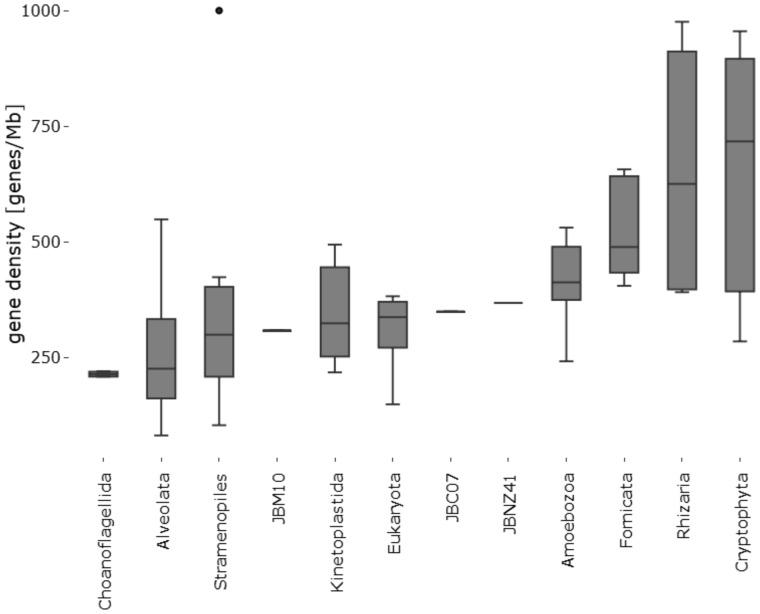
—Gene density. Comparison of *Poteriospumella lacustris* against 197 protists from the Ensembl Protists database.

The gene density was roughly 310–370 genes/Mb. In comparison to other protists, *P. lacustris* is among the species with the lowest gene densities, but with a comparable density to related species in the group of stramenopiles (see fig. 2).

The gene prediction was evaluated with the respective transcriptome, but the RNA sequences could not be used to train and create a specific model for *P. lacustris*. Despite this, the data could be used to improve an existing model and for validation and accuracy checking. Applying the prediction model of *A.thaliana* 81–84% of RNA reads could be mapped back to the genome. Improving the model with transcriptomic data enhanced the mapping by 0.16%. In total, 55,941 (JBC07), 60,147 (JBM10), and 62,248 (JBNZ41) genes were predicted. By pooling the predicted genes and clustering we obtained a total amount of 47,500 unique genes, of which 25,693 genes could be annotated. This procedure merged duplicates and gene variants. The pooled genes were aligned back to each genome (sensitivity > 99%, see fig. 1), resulting in the following numbers of genes: The genomes of JBC07, JBM10, and JBNZ41 contained 17,315, 16,915 and 19,494 genes, respectively, of which 7,453, 7,960, and 8,525 were annotated with KEGG identifiers.

**Fig. 3.**
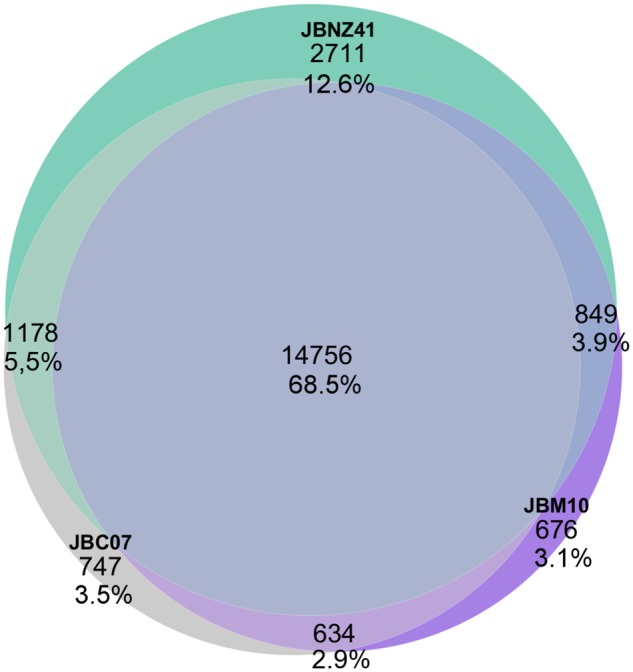
—Venn diagram. Proportion of shared genes. Percentages refer to the total number of 21,551 genes.

All three strains combined contained 21,551 genes. 14,756 genes (68.5%) were present in every strain (see fig. 3). 80.8% of the genes were shared with at least one other strain. The proportions of genes found in a single strain were 3.5% (JBC07), 3.1% (JBM10), and 12.5% (JBNZ41). Shared genes had a sequence similarity of 97.3% and 0.0018 deletions/inserts per gene on average.

### Ploidy Estimation

**Fig. 4.**
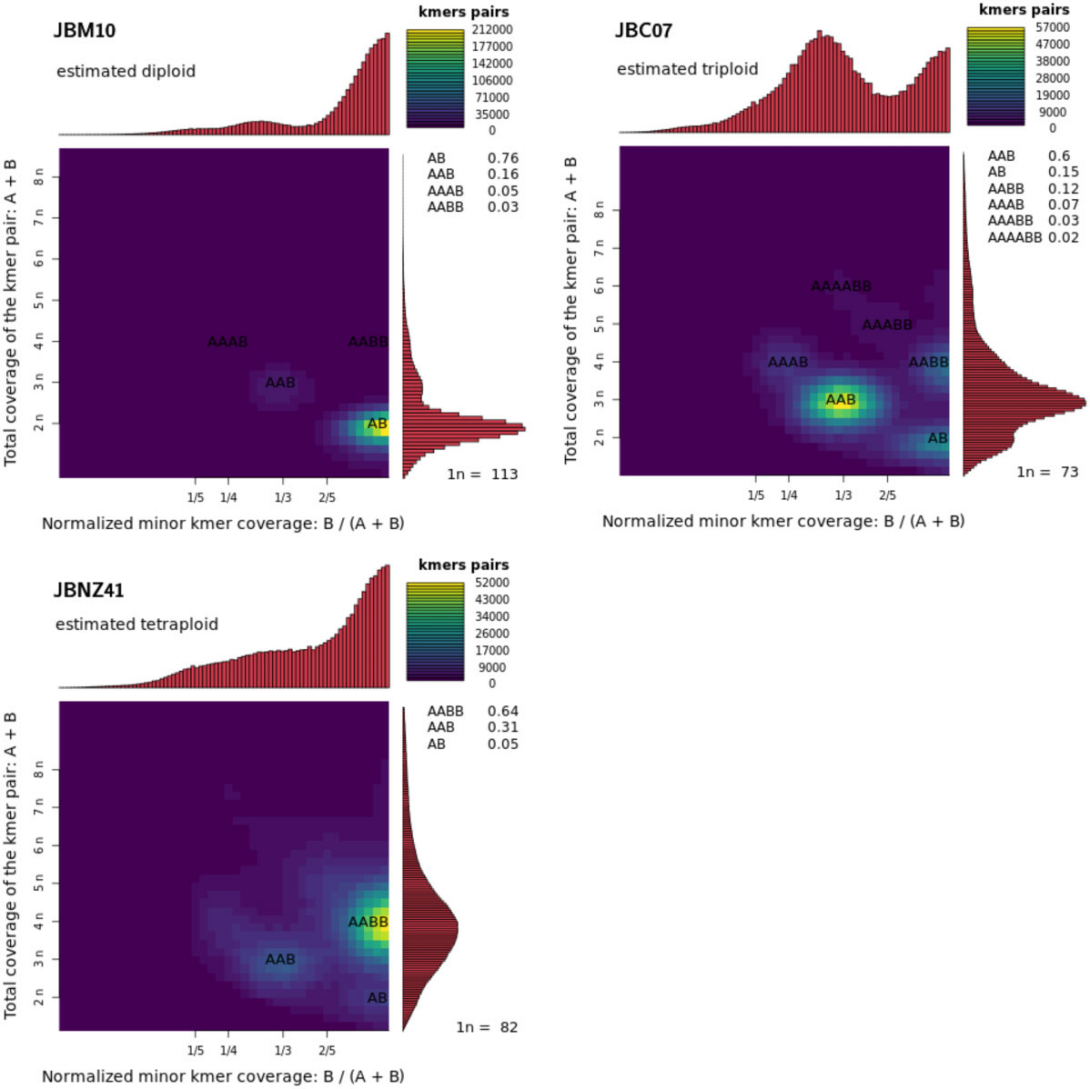
—*K*-mer based ploidy estimation. The heatmap reflects coverage of *k*-mer pairs differing by one base. The ratio of the characters A to B represents the ratio of these *k*-mer pairs (e.g., 67% ATGTC and 33% ATGTT conforms AAB). The coverage distribution on the right side indicates ploidy levels (left scale). The distribution on the top side is based on the coverage normalized by the ratio. The determined ploidy levels of the strains were diploidy (JBM10), triploidy (JBC07), and tetraploidy (JBNZ41). *n* = average *k*-mer coverage, *k*-mer size = 21.

Ploidy estimation indicates triploidy in JBC07, diploidy in JBM10 and tetraploidy (or diploidy) in JBNZ41 based on the SNPs of the denoised data set as well as on the data set with excluded repeats (details see supplementary tables S4, S5 and fig. S2, Supplementary Material online). For the ploidy estimation on single contigs each data set consisted of 850–934 contigs. The criteria of a >10% higher probability of a certain ploidy was fulfilled for 78–79% (JBM10, JBNZ41) and 65% (JBC07) of the contigs. Estimating the ploidy for individual contigs of JBC07, the majority of the contigs (45%) was triploid, but also a high proportion of contigs (39%) were rated diploid (see supplementary S4, Supplementary Material online). The *k*-mer based approach approved the estimated ploidy levels (JBC07: triploid, JBM10: diploid, and JBNZ41: tetraploid; see fig. 4). The variation in ploidy level was unexpected and shows that the intraspecific molecular differences were higher than initially assumed.

### Variant Analysis

**Fig. 5.**
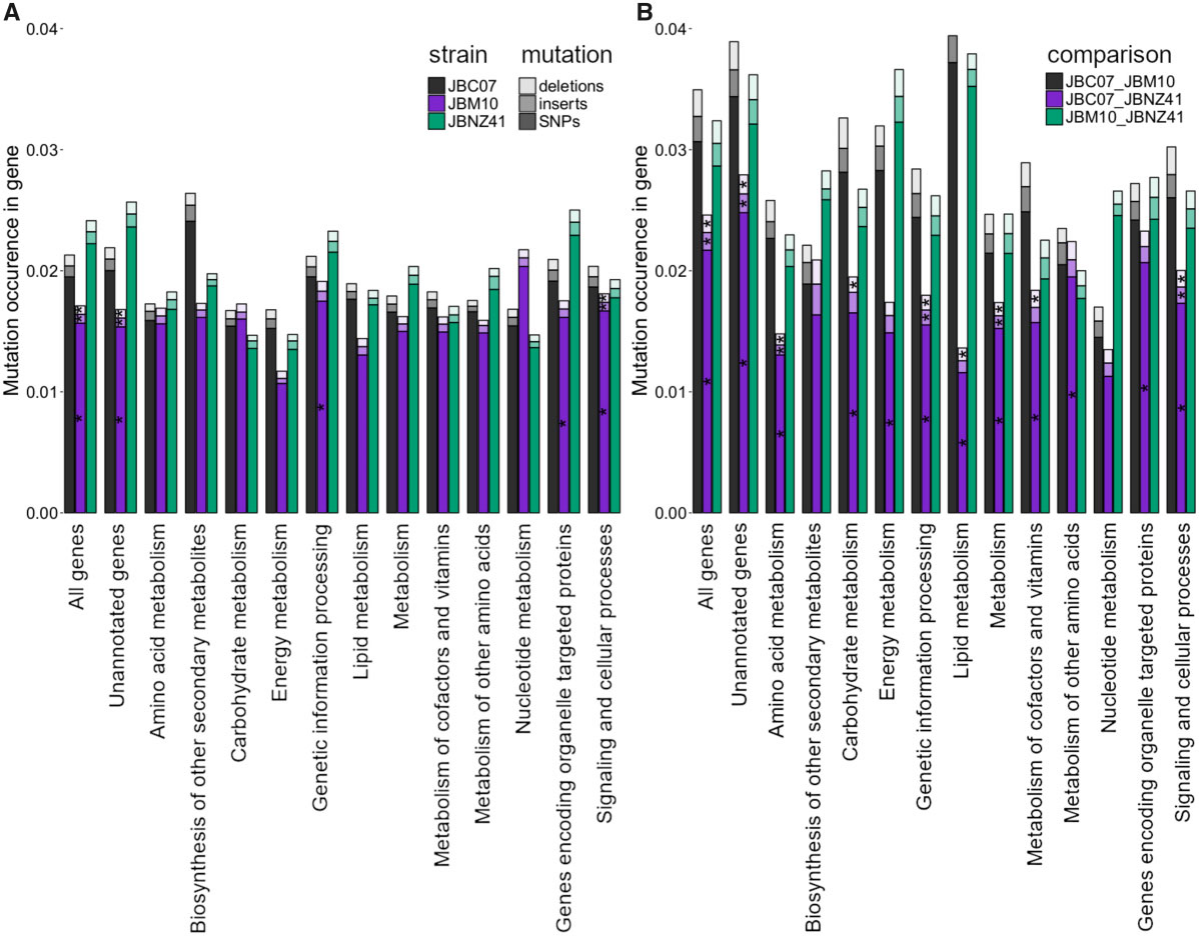
—Allelic variation and mutation distribution. Occurrence of SNPs (dark), insertions (semitransparent), and deletions (bright) in the strains JBC07 (gray), JBM10 (purple), and JBNZ41 (green). The significance (*P*-value < 0.01) within one functional group was calculated by ANOVA. The asterisk was placed in the middle column (JBM10) and refers to one mutation type within the three strains. (*A*) The distribution of mutations displays the allelic variation within one strain. There the energy metabolism is most conserved. (*B*) Pairwise comparison of genes demonstrates which groups evolved apart and which stay conserved. Especially JBM10 deviates from the other two strains.

Separating the SNPs, inserts and deletions according to metabolic pathway categories showed differences in their mutation rate. Likewise, the strains differ in the mutation rate within a metabolic pathway category. We examined if mutation rates of a specific functional group differed between strains (ANOVA or Kruskal–Wallis test, see supplementary tables S12 and S13, Supplementary Material online) and if mutation rates of a specific strain differed between functional groups (Wilcoxon signed-rank test, see supplementary tables S6–S11, Supplementary Material online). We first enumerated the allelic variation of each strain (see fig. 5*A*, see supplementary tables S6–S8, Supplementary Material online). The strain JBM10 had except for the category *nucleotide metabolism* a significantly (*P* < 0.01) lower variation rate than JBC07 or JBNZ41 (see fig. 5*A*, ANOVA see supplementary table S12, Supplementary Material online). Molecular variation between the strains differed significantly in the categories *unannotated genes, genetic information processing, organelle targeting genes* and *signaling and cellular processes* (see supplementary table S12, Supplementary Material online). Molecular variation between the functional groups of each strain was significant (*P* *<* 0.01) for: *genetic information processing* (all strains), energy metabolism (JBM10, JBNZ41), *unannotated genes (*JBM10, JBNZ41)*, signaling and cellular processes* (JBM10), and *carbohydrate metabolism* (JBNZ41) (see supplementary tables S6–S8, Supplementary Material online).

A pairwise comparison of genes between different strains demonstrated genetic divergence (see fig. 5*B*, statistic see supplementary tables S9–S11, Supplementary Material online). Across nearly all categories JBM10 deviated from the other two strains. Exceptional categories were *biosynthesis of other secondary metabolism* and *nucleotide metabolism*, which showed no significant difference (see supplementary table S13, Supplementary Material online). In the pairwise comparison between strains the group of *unannotated genes* differed the most (see supplementary tables S9–S11, Supplementary Material online). Moreover, ∼1,000 identical genes were shared between JBC07 and JBNZ41, whereas JBM10 had 584 (JBC07) and 430 (JBNZ41) identical genes in common (see supplementary table S3, Supplementary Material online).

**Fig. 6.**
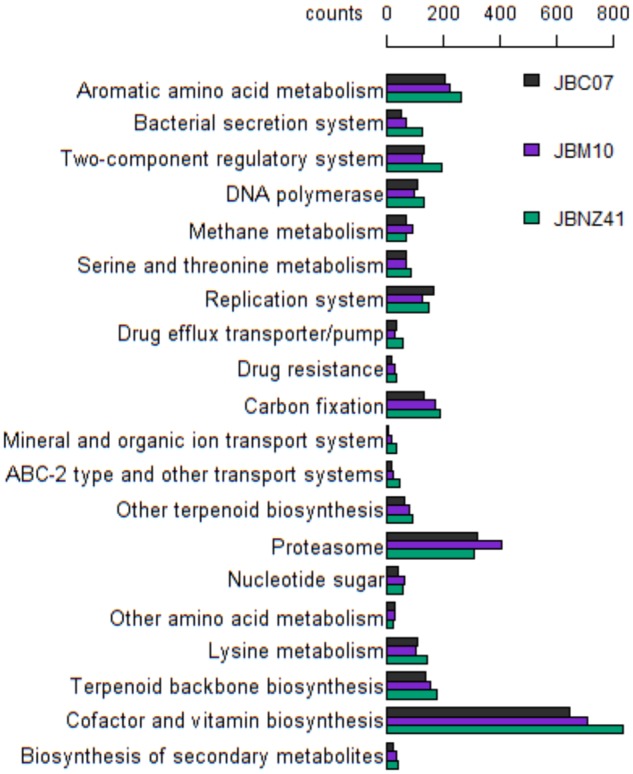
—Gene count based on the KEGG hierarchy functional assignment. Only functional categories with at least 20 counts and >10% standard deviation between the strains are shown.

Counting genes based on the KEGG functional assignment showed small differences between the strains (see fig. 6). Notably, JBM10 contains more genes assigned to *proteasome* and JBNZ41 comprises more genes of *cofactor and vitamin biosynthesis* compared with the other two strains.

The *genes encoding organelle targeted proteins* identified by HECTAR were subdivided in the groups signal peptide, signal anchor, mitochondrion and chloroplast. The associated mutation rate between these classes, however, did not differ significantly (*P* > 0.05), which is why we kept the combined group *genes encoding organelle targeted proteins.*

The ANI was between 97.4% and 98.0% (see table 2). Despite the high similarity of the entire genome, we could identify distinctions between primary and secondary metabolism. The number of SNPs in pathways of the secondary metabolism was generally higher (see fig. 5*B* and supplementary tables S9–S11, Supplementary Material online). However, the SNPs gave us insufficient information about recent population development due to higher variation caused by different ploidies. The mitochondrial DNA was almost identical (one insertion in JBM10 and 1–5 SNPs between each strain, despite ∼38,000 bp length).

## Discussion

### Genome Assembly and Gene Prediction

Genome comparison of three *P.lacustris* strains (JBM10, JBNZ41, JBC07) revealed similarities in genes and their structural arrangement. Although the strains were assembled based on Illumina and PacBio sequencing, the assembly size of the genomes deviated from the estimated sizes based on nuclear staining (Olefeld et al. 2018) (see table 1). This discrepancy may indicate different ploidy levels which were not considered by Olefeld et al. (2018) (see discussion on ploidy below). Additionally, the high number of contigs in the genome assemblies reflected the extent of fragmentation. It is clearly a challenge to assemble large and possibly repetitive eukaryotic genomes and new technologies such as MinION sequencing with an even larger read length could possibly enhance the genome assemblies. Nevertheless, the amount of contigs was comparable to other eukaryotic hybrid assemblies (e.g., *Aegilops tauschii*: *>*24,000 contigs [Zimin et al. 2017], *Melopsittacus undulatus*: *>*15,000 contigs [Ganapathy et al. 2014]).

The BUSCO analysis could not answer the question of genome completeness adequately since the comparative gene set was unsuitable for *P. lacustris*. In nonmodel microbial eukaryotes the model gene sets used in BUSCO or CEGMA could be limited due to distantly related model organisms as was shown for example in dinoflagellates of the genus *Symbiodinium* (Aranda et al. 2016; Liu et al. 2018).

The completeness of essential primary metabolic pathways (citrate cycle, pentose phosphate pathway, nucleotide and protein biosynthesis, etc.) in our analyses, assessed with the KEGG Mapper—Reconstruct Module tool, affirmed genome integrity. Furthermore, we found genes affiliated with phototrophic pathways as well as pathways related to autotrophy (e.g., sulfur assimilation), which are presumably remainings from the phototrophic ancestor (Beisser et al. 2017; Graupner et al. 2018). More evolved heterotrophs have lost these pathways, providing evidence that *P. lacustris* is in an early stage of heterotrophy (Graupner et al. 2018). The reduction of phototropic pathways confirms the natural selection and adaptation of *P. lacustris*.

Since our gene analysis is based on an unclosed genome it could naturally lead to errors, especially with respect to gene counts (Denton et al. 2014). Therefore, we reduced the number of predicted genes by clustering and marking of duplicated genes if they overlapped on the same strand in the alignment between genes and genome. As a result, the total amount of 178,000 genes decreased to around 18,000 genes in each strain (see fig. 1). The approach of merging overlapping genes may, lead to a loss of nested genes. This loss is neglect able since the number of nested genes of different strands in eukaryotic genomes is very small (0.7–0.8% of the total amount of genes, Sanna et al. 2008). The gene level is slightly lower than the estimated ∼20,000 genes found in the transcriptome study of *P.lacustris* (Graupner et al. 2017). However, the gene number in the study of Graupner et al. (2017) was based on gene components generated by the Trinity software, which could therefore be higher because of isoforms and variants of genes.

### Gene Density

Even though the genome sizes of heterotrophic chrysomonads is smaller than in mixotrophic or phototrophic relatives (Olefeld et al. 2018), the gene density remains at a comparable level to other protist species, even compared with predominantly phototrophic relatives (see fig. 2). It is assumed that heterotrophic chrysophytes have a selection pressure toward small cell sizes, which enable more effective preying on ultra small bacteria (Hansen 1992; Hansen et al. 1994; Olefeld et al. 2018). Deletions of noncoding DNA sequences are likely mechanisms to decrease the genome size. The gene density varies between the strains due to differences in gene length. However, the average ratio of coding DNA in proportion to total DNA is consistent, although there are small differences in the proportion of repeat regions (see table 1).

### Ploidy

Surprisingly, ploidy levels differed between the strains despite their close relatedness. Earlier attempts to stain the chromosomes for microscopic ploidy assessment failed because of the small nucleus and chromosome size of *P. lacustris*. By using flow cytometry, it has been shown that the genome size of JBM10 is distinctly smaller than those of the other two strains (Olefeld et al. 2018). This method assumes equal ploidy and can only determine the total amount of DNA. On the basis of the genome assembly the haploid genome size is ∼50 Mb for all three strains and from the molecular data, we have indications that the grade of ploidy differs between the three strains. Allelic distribution of SNPs suggests diploidy in JBM10, triploidy in JBC07, and tetraploidy in JBNZ41. This is in accordance with the genome size estimates of (Olefeld et al. 2018)—the genome of JBNZ41 is approximately twice as large as that of JBM10 and the genome size of JBC07 is in-between (see table 1). However, as we found only two allelic variants for numerous genes we assume that JBNZ41 became tetraploid with a recent genome duplication. This would explain the strong peak indicating diploidy for many genes with weaker (even though pronounced) peaks indicating tetraploidy for other genes and at the same time the larger genome size (see supplementary fig. S1, Supplementary Material online).

Tetraploid strains with a characteristic diploid distribution were also reported for *Saccharomyces cerevisiae* (Zhu et al. 2016). The ploidy estimation based on kmers, which is independent of possible assembly biases approved our results (see fig. 4). Nevertheless, all strains showed at least partial triploid-like peaks. This could be induced by a large number of paralogous genes or gene duplications only on one locus. Polyploidy has also been found in some other relatives of the stramenopiles, for example, in diatoms (Parks et al. 2018), oomycetes (Martens and Van de Peer 2010), and brown algae (Cock et al. 2010). Together with the genome size data from (Olefeld et al. 2018) our data provide evidence for different levels of ploidy in closely related strains. Nonetheless, the presumable genome duplication contradicts the hypothesis of a strong selection pressure toward small cell sizes and small genomes (de Castro et al. 2009; Olefeld et al. 2018) indicating that other factors beyond predator–prey interactions may also be significant in the genome evolution of heterotrophic chrysophytes.

### Variation between Strains

The larger genome size of related mixotrophic taxa indicate that *P.lacustris* reduced the genome size as it developed a heterotrophic mode of nutrition. The decreased genome size but constant gene density implies a loss of genes. In general, mutations already occur, before a gene gets lost or the gene function is changing. Further, after a gene lost its function, mutations appear more frequently. Consequently, genes with more mutations are potentially less important for an organism or several copies may exist. Organisms with a higher ploidy often show higher mutation frequencies (e.g., Ohnishi et al. 2004; Uauy et al. 2009; Krasileva et al. 2017), because they have multiple genes as back up or several gene variants for special conditions. Therefore, we compared the variation of genes grouped by their function (see fig. 5*A*). Some groups (e.g., *biosynthesis secondary metabolism*) seemed to have a high variation, but differences were nonsignificant due to the high standard deviation of mutation occurrence. In all three strains the category of *genetic information processing* contained the most highly variable genes and, in contradiction, also the highest proportion of identical genes between the strains (see supplementary table S3, Supplementary Material online). Apparently, *genetic information processing* is vital. The high number of mutations in genes affiliates with this pathway may indicate that this system is manifold fail-safe so that mutations presumably can be tolerated without severe consequences. In JBM10, noticeably many mutations occurred in the group of *cellular processes* belonging predominantly to *cGMP signaling, DNA damage-induced cell cycle checkpoints, MAPK signaling* and *Cell cycle—G2/M transition*. The variation in the MAPK pathway may indicate decreased environmental stress including osmotic and thermal changes (Jimenez et al. 2004). Variances in *cell cycle supervision* may have led to a higher proliferation rate or cell death (Hartwell and Weinert 1989; Stark and Taylor 2006). However, the growth rate of *P. lacustris* is comparable to related species like *Poterioochromonas malhamensis* or *Dinobryon divergens* (Rottberger et al. 2013). Like in the category *genetic information processing*, the genes affiliated with *cellular processing* are present in multiple copies. On the other hand, genes assigned to energy metabolism comprise fewer variations possibly indicating that fewer mutations are acceptable. In general JBM10 has a lower allelic variation than JBC07 or JBNZ41. Since JBM10 also has a smaller genome size (Olefeld et al. 2018), but a similar gene density (see table 2), this strain consequently possess fewer gene copies and therefore less allelic variation. Especially, higher ploidy would enable higher recombination rates (Song et al. 1995). In addition, polyploids comprise significantly more allelic variation than diploids (Li et al. 2017), which may explain for higher variation within JBC07 and JBNZ41.

We performed a pairwise comparison for each gene shared between strains to count mismatches between two genes if no allelic variation could induce an identical sequence. This enabled us to detect possibly identical genes within the alleles between strains as well as the genetic variation (see supplementary table S3, Supplementary Material online). The genes of strains JBC07 and JBNZ41 are genetically more similar to each other than to JBM10 (see fig. 5). This indicates a closer relation between JBC07 and JBNZ41, which also originate from geographically closer sites. It must be noted, that with increasing allelic variation the probability for random matches also increases. In other words, the probability that the same alleles are found between a triploid and a tetraploid organism is higher than when compared with a diploid strain. Thus, allelic variation could not be clearly assigned to either differences in ploidy or phylogenetic relatedness.

Most mutations occurred in the set of genes without a functional group assignment (see fig. 5*B*). One reason could be a false positive gene interpretation during the prediction procedure (noncoding sequence interpreted as unknown gene). It can be assumed that noncoding DNA presumably has a weaker selection pressure and therefore more mutations (Andolfatto 2005). The KEGG database relies on genes assigned with a function. These information are gained from well-known organisms which are not closely affiliated with our target strains but with other supergoups. Possibly the annotated *P. lacustris* genes could have only the essential genes in common with these model organisms. Hence, the other (unassigned) genes might be necessary for ecological niche adaptation or species-specific functions, but not for key primary metabolic pathways. Our data suggest that the secondary metabolism in *P. lacustris* is subject to stronger genetic changes than the primary metabolism.

The number of shared genes (see fig. 3) reflects the close relationship between the three strains. In contrast to the indications from sequence variation discussed above, the strains JBC07 and JBM10 have more genes in common, whereas JBNZ41 has the highest number of strain-specific genes indicating a more distant relation. This constellation of the relationships was also described in Beisser et al. (2017) and Stoeck et al. (2008), but contradicts the findings of Graupner et al. (2017) based on orthologous genes. The three strains share 68.5% of all genes. However, the overlap of shared genes could rise when the genome sequences are completed. On the other hand, Graupner et al. (2017) determined around 92% annotated shared genes (*n* = 2,000) and 50% sequences variations in general (*n* = 20,000), which confirms our results. *Poteriospumellalacustris* has a slightly less gene diversity as the phytoplankton *Emiliania huxleyi* (75% shared genes; Read et al. 2013) and more than the fungus *Zymoseptoria tritici* (58% shared genes; Plissonneau et al. 2018). Because of the high number of shared genes, it is not surprising that the gene count varies little by function (see fig. 6).

The extent of variation in the mitochondrial sequences should be similar or even larger than the variation between the genomes (Smith 2015). However, the mitochondrial DNA remains conserved. This findings accord to phylogenetic analyses of COI genes within chrysophytes, where 18 strains of *P. lacustris* clustered together (Bock et al. 2017). However, the conserved mDNA could not be generalized within chrysophytes since other species varied in the COI gene (Bock et al. 2017). Furthermore, there are some other species showing very low intraspecifc mitochondrial variation (like the coral Octocorallia, McFadden et al. 2010). In comparison *genes encoding organelle targeted proteins* have similar mutation rates to all other genes (see supplementary tables S6–S11, Supplementary Material online).

Subsequent studies should include further species to cover the whole class of Chrysophyceae and especially include representatives with phototrophic and mixotrophic nutrition in order to shed light on the genome evolution in the course of the multiple parallel nutritional adaptation from mixotrophy to heterotrophy as well as species of different ploidy levels to consider the ploidy as an influencing variable. Further, a more extensive analysis of transposons could extend the analysis of genome evolution in Chrysophyceae.

## Conclusions

Our study provides a comprehensive genome analysis and created one of the first reference genomes within the Chrysophyceae. The intraspecific genome variation of *P. lacustris* is high, especially the level of ploidy. Most mutations occurred in unannotated genes, which are likely related to secondary metabolism and to the adaption to a particular niche. We thus can reject the hypothesis that mutations are randomly distributed across pathways and metabolic categories. Since all strains differ in the degree of ploidy, it was not possible to deduce past population bottlenecks based on the allelic variation.

## Supplementary Material

Supplementary data are available at *Genome Biology and Evolution* online.

## Supplementary Material

evz171_Supplementary_Data

## Notes

**Data deposition:** This project has been deposited at NCBI under the accessions PRJNA504602, PRJNA504603, PRJNA504604.
