# Supplementary material for: Intraspecific Variation in Protists: Clues for Microevolution from Poteriospumella lacustris (Chrysophyceae)
Source: Genome Biol Evol. 2019 Aug 6;11(9):2492–504. doi: 10.1093/gbe/evz171 (PMC6738136; doi:10.1093/gbe/evz171)
Supplement: evz171_Supplementary_Data [file evz171_supplementary_data.pdf]

Supplement to the paper: intraspecific variation in protists: clues for microevolution from *Poteriospumella lacustris* (Chrysophyceae)

Detailed Methods

Gene Prediction

The gene prediction was tested in different ways:
(1) At the first place, Tophat2 (Kim et al., 2013) combined with GeneMark-ET (Borodovsky and Lomsadze, 2011) was applied to train the gene prediction with RNA-Seq data (Beisser et al., 2017). Subsequently, the tool AUGUSTUS (Stanke et al., 2006) was used for gene prediction. To verify the prediction results, we mapped the RNA reads to the predicted genes with Bowtie (v.2.2.8 with parameters: –very-sensitive-local; Langmead et al., 2009). This attempt was discarded, because of the low back mapping rate (65-70%)
(2) To create a species-specific prediction model, we followed the instructions on http://augustus.gobics.de/binaries/retraining.html with RNA-Seq data (Beisser et al., 2017). It was not achievable to create a sufficient *P. lacustris* specific model with the current data. Therefore, the gene pattern of *Arabidopsis thaliana* was chosen as model instead.
(3) AUGUSTUS can improve the prediction model with additional information on expressed sequence tag (EST). Because the transcriptomic reads (Beisser et al., 2017) were not satisfactory, we used known algal sequences. Therefore, algae sequences of Alga-PrAS database (`http://alga-pras.riken.jp/menta.cgi/static/algapras/Alga-PrAS\_Resource.zip;retrieved12/2017`) were aligned with the assembled genome of strain JBM10 by Exonerate (v2.2.0 with parameters: –model protein2genome; Slater and Birney, 2005). The alignment of about 500,000 possibly present protein sequences determined potential introns, which where masked with BEDTools (v2.27, with parameters: maskfasta -bed GFF; Quinlan and Hall, 2010). RNA reads of the

strains (Beisser et al., 2017) were mapped to the masked genome. Thereby, gene occurrence was confirmed and consensus sequences of the alignment were declared as EST. In a trial run a file with the ESTs was used to support the gene prediction of AUGUSTUS.
(4) Finally, we used AUGUSTUS (v3.3 with parameters: –species=arabidopsis –gff3=on –singlestrand=true –UTR=off; Stanke et al., 2006) for gene prediction. Because of the minimal benefit (e.g. 0.16% enhancement of the back mapping rate) of attempt (3) to (4) and the expensive computational calculations we favored (4).
(5) Retrospectively, we compared (4) with an additional approach. The RNA Seq data (Beisser et al., 2017) was aligned with the genome by Minimap2 (2.16-r922; with parameters: -c -L -x splice -G 80K -t 16). The tool RepeatScout (v1.0.5; Price et al., 2005) provided repeat sequences, which were used to mask the genome with BEDTools (v2.28, with parameters: maskfasta -soft). Afterwards, AUGUSTUS (v3.3 with parameters: –softmasking=1 –species=arabidopsis –gff3=on –singlestrand=true –UTR=off –alternatives-from-evidence=false –extrinsicCfgFile –hintsfile) was used to predict the genes. In the extrinsicCfgFile the following relevant weightings were set:
exon hit bonus: 1e+10 (instead 1), nonexonpart malus: 2 (instead 1)
Genes of attempt (4) and (5) were clustered with CD-HIT (v4.7 with parameters: cd-hit-est -c 0.85 -s 0.7; Li and Godzik, 2006). Both methods share 94% of predicted genes (mean identity= 98.9, standard deviation 3.0). Approach (5) predicted 2% less genes. Methods (4) and (5) generated comparable results within the usual variation between gene prediction approaches.

Literature Cited

Beisser D, et al. 2017. Comprehensive transcriptome analysis provides new insights into nutritional strategies and phylogenetic relationships of chrysophytes. PeerJ 5:e2832.

Borodovsky M, Lomsadze A. 2011. Eukaryotic gene prediction using genemark.hmm-E and genemark-ES. Curr Protoc Bioinformatics. 35: 4.6.1–10.

Kim D, et al. 2013. Tophat2: accurate alignment of transcriptomes in the presence of insertions, deletions and gene fusions. Genome Biol. 14(4):R36.

Langmead B, et al. 2009. Ultrafast and memory-efficient alignment of short DNA sequences to the human genome. Genome Biol. 10(3):R25.

LiW, Godzik A. 2006. Cd-hit: a fast programfor clustering and comparing large sets of protein or nucleotide sequences. Bioinformatics 22(13):1658–1659.

Price AL, et al. 2005. De novo identification of repeat families in large genomes. Bioinformatics 21(Suppl 1):351–358.

Quinlan AR, Hall IM. 2010. BEDTools: a flexible suite of utilities for comparing genomic features. Bioinformatics 26(6):841–842.

Slater GSC, Birney E. 2005. Automated generation of heuristics for biological sequence comparison. BMC Bioinformatics. 6(1):31.

Stanke M, et al. 2006. AUGUSTUS: ab initio prediction of alternative transcripts. Nucleic Acids Res. 34(Web Server issue):435–439.

Table S1: ***K*-mer based genome size estimation**
GenomeScope (version 1.0; with parameters: k = 21, read = 150, max cov. 10,000; http://qb.cshl.edu/genomescope/)

JBC07

property	min	max
Heterozygosity	0.526297%	0.558878%
Genome Haploid Length	73,203,643 bp	73,946,828 bp
Genome Repeat Length	61,182,149 bp	61,803,288 bp
Genome Unique Length	12,021,494 bp	12,143,539 bp
Model Fit	74.7753%	76.031%
Read Error Rate	0.458844%	0.458844%

JBM10

property	min	max
Heterozygosity	1.84751%	1.86612%
Genome Haploid Length	38,945,278 bp	38,994,499 bp
Genome Repeat Length	11,944,582 bp	11,959,679 bp
Genome Unique Length	27,000,695 bp	27,034,821 bp
Model Fit	90.1891%	91.2805%
Read Error Rate	0.487495%	0.487495%

JBNZ41

property	min	max
Heterozygosity	0.395286%	0.406731%
Genome Haploid Length	69,026,950 bp	69,267,407 bp
Genome Repeat Length	51,677,816 bp	51,857,836 bp
Genome Unique Length	17,349,135 bp	17,409,571 bp
Model Fit	85.7327%	90.24%
Read Error Rate	0.450912%	0.450912%

Table S2: **BUSCO genome completeness check.** The data sets for eukaryotes and protist were used to check the genome integrity. Additionaly, the genomes of *Nannochloropsis oceanica* and *Ectocarpus siliculosus* (from NCBI) were used as comparison.

BUSCO set	*P. lacustris* (JBC07)	*P. lacustris* (JBM10)	*P. lacustris* (JBNZ41)	*Nannochloropsis oceanica*	*Ectocarpus siliculosus*
EUK set (303 BUSCO groups)					
Complete [%]	79.2	79.9	75.2	78.9	77.6
Complete and single-copy [%]	68.6	70.0	70.6	76.9	76.6
Complete and duplicated [%]	10.6	9.9	4.6	2.0	1.0
Fragmented [%]	4.6	3.6	6.3	5.5	6.9
Missing [%]	16.2	16.5	18.5	15.8	15.5
protist set (215 BUSCO groups)					
Complete [%]	55.8	56.3	53.5	63.3	68.9
Complete and single-copy [%]	45.6	51.2	51.2	63.3	68.4
Complete and duplicated [%]	10.2	5.1	2.3	0.0	0.5
Fragmented [%]	0.0	0.0	0.9	0.9	0.9
Missing [%]	44.2	43.7	45.6	35.8	30.2

Table S3: **Identical genes.** Number of genes that are 100% identical in at least one allele between two strains. Only functional groups containing more than 40 genes are listed. JBC07 and JBNZ41 share about twice as many identical genes as they share with JBM10. The group of genetic information processing and metabolism, mainly enzymes, are highly conserved between all strains.

KEGG functional hierarchy L1	JBC07 JBM10	JBC07 JBNZ41	JBM10 JBNZ41
All genes	584	1041	430
Unannotated genes	356	717	242
Amino acid metabolism	6	8	13
Biosynthesis of other secondary metabolites	0	1	0
Carbohydrate metabolism	6	3	3
Energy metabolism	2	10	6
Genetic information processing	92	126	53
Lipid metabolism	2	1	1
Metabolism	52	52	39
Metabolism of cofactors and vitamins	8	7	7
Metabolism of other amino acids	4	9	4
Nucleotide metabolism	0	4	1
Signaling and cellular processes	38	67	33

Table S4: **Ploidy estimation for each contig.** Quantity of contigs assigned with ploidy level and normalized by contig length. Only contigs longer than 10 kbp were counted and used, if ploidy assessment value differ at least 10% from the other ploidy estimations. Recent tetraploidy could also be assigned to diploidy in contigs with insufficient diverging SNPs.

Ploidy	JBC07	JBM10	JBNZ41
diploid	84.0	**184.1**	**172.3**
triploid	**97.2**	41.1	81.3
tetraploid	33.6	48.7	48.1
percentage used contigs	65	79	78

Table S5: **Ploidy estimation of the nQuire tool.** Columns free, dip (diploid), tri (triploid) and tet (tetraploid) show log values of model probabilities. Rows starting with "d_" indicate the divergence to the real model. The model with the smallest deviation determine the ploidy level."

file	JBC07_denoised.bin	JBM10_denoised.bin	JBNZ41_denoised.bin
free	253,438.79	567,363.69	308,681.32
dip	73,869.62	537,303.73	292,502.65
tri	204,201.79	167,745.84	123,628.44
tet	78,299.84	330,984.04	164,817.24
d_dip	179,569.18	**30,059.95**	**16,178.66**
d_tri	**49,237.01**	399,617.85	185,052.88
d_tet	175,138.95	236,379.65	143,864.08

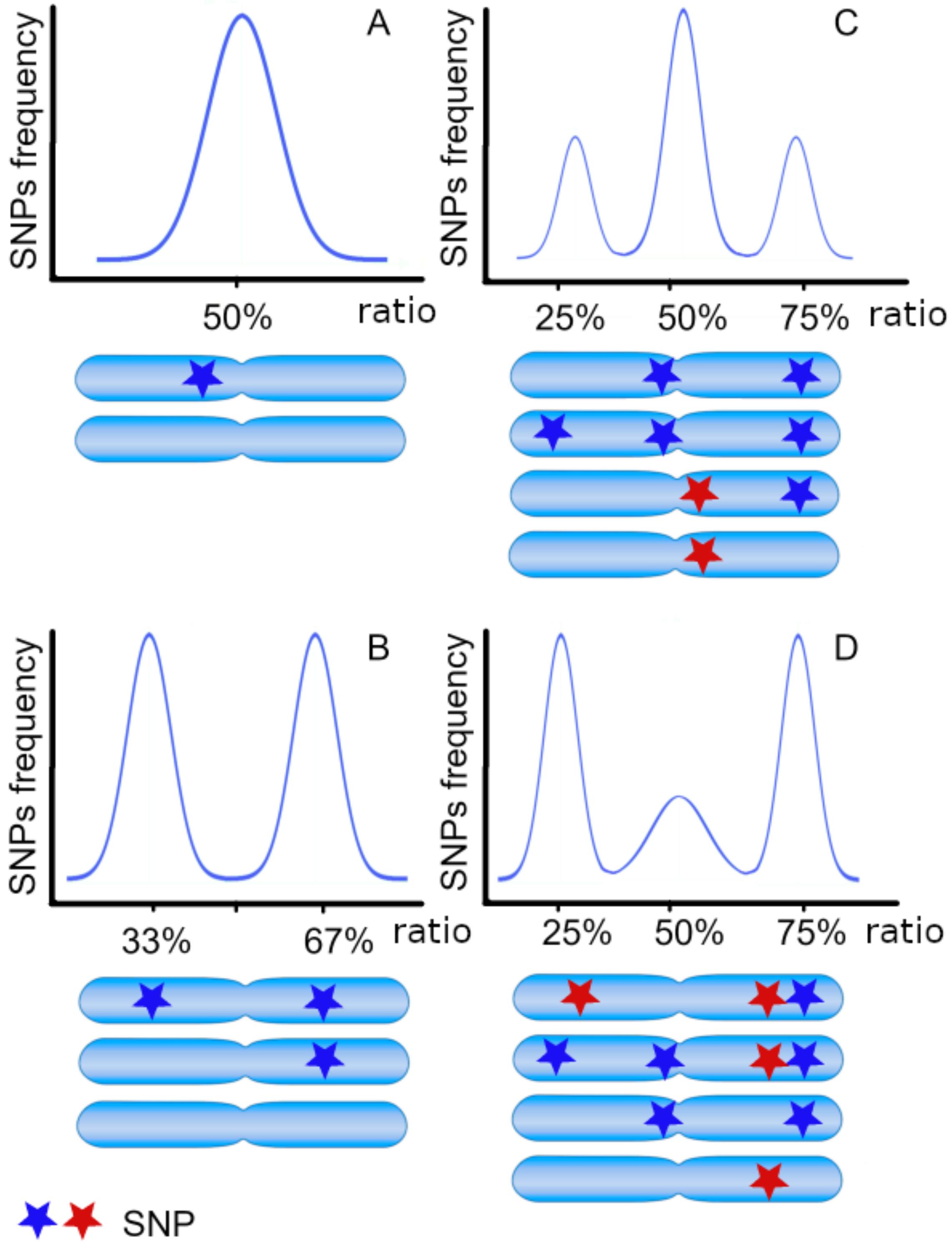

Figure S1 Schematic of SNPs based ploidy estimation. The ratio of alleles with the SNPs (red and blue stars) to alleles without indicates the ploidy level. The *P. lacustris* strains showed distributions associated with diploidy (**A**, JBM10), triploidy (**B**, JBC07) and recent tetraploidy (**C**, JBNZ41), whereas ancient tetraploidy (**D**) was not found. A recent tetraploidy organism has a shift of the majority in SNP frequencies to 50% as it arises from the doubling of a diploid genome. The red stars indicate the majority ratios.

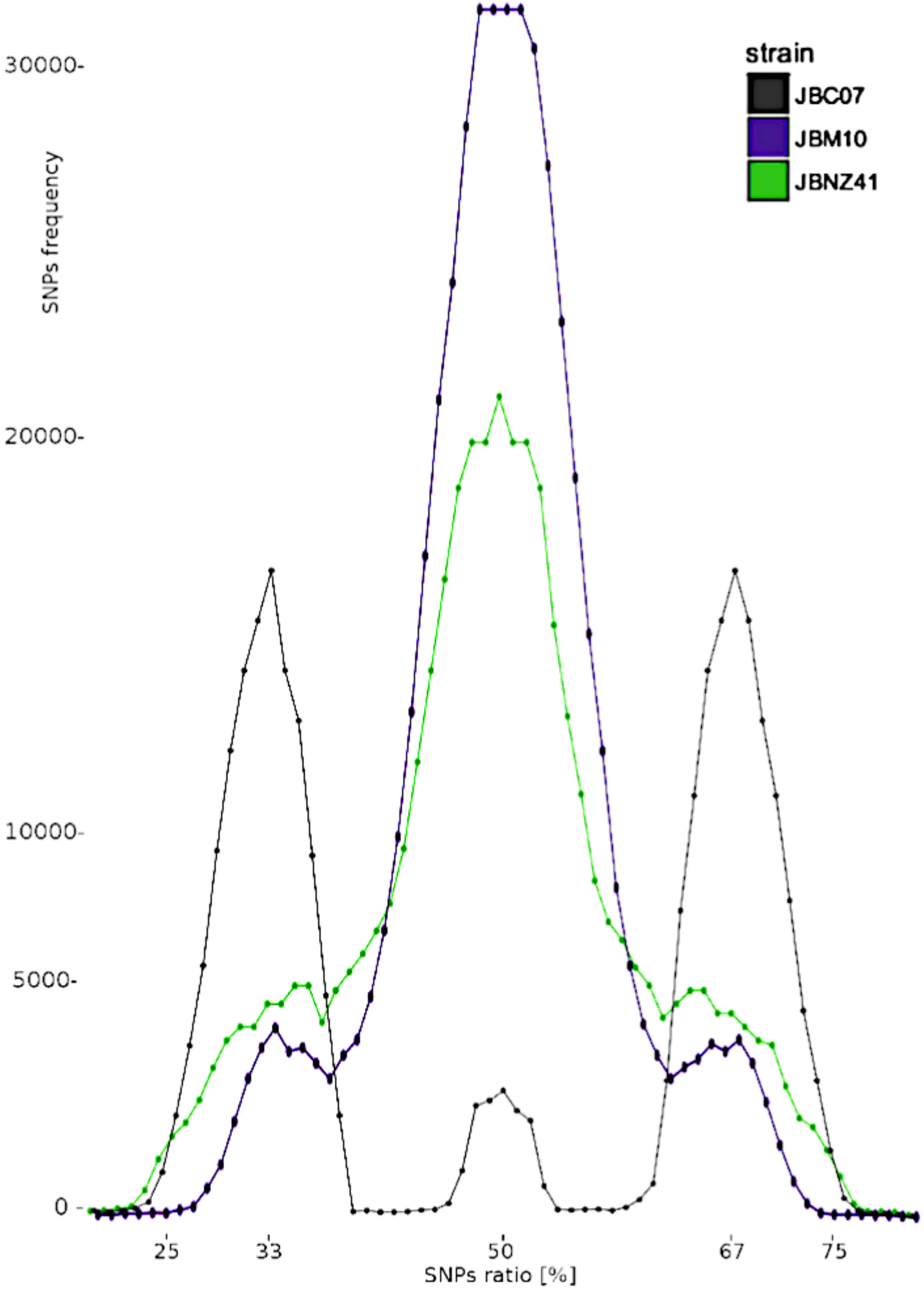

Figure S2 Ploidy estimation based on SNPs. The ratio of SNPs correlates with the ploidy. The determined ploidy levels of the strains were diploidy (JBM10), triploidy (JBC07) and diploidy or tetraploidy (JBNZ41).

Table S6, S7 and S8: Strain specific Wilcoxon signed-rank tests on gene variation within functional groups validate pairwise differences. P-values (< 0.01) were bold.

JBC07

	All genes	Unannotated genes	Amino acid metabolism	Biosynthesis of other secondary metabolites	Carbohydrate metabolism	Energy metabolism	Genetic information processing	Lipid metabolism	Metabolism	Metabolism of cofactors and vitamins	Metabolism of other amino acids	Nucleotide metabolism	Organelle targeting genes
Unannotated genes	8.8E-01	NA	NA	NA	NA	NA	NA	NA	NA	NA	NA	NA	NA
Amino acid metabolism	5.1E-01	5.4E-01	NA	NA	NA	NA	NA	NA	NA	NA	NA	NA	NA
Biosynthesis of other secondary metabolites	4.6E-01	4.6E-01	2.3E-01	NA	NA	NA	NA	NA	NA	NA	NA	NA	NA
Carbohydrate metabolism	5.1E-01	5.4E-01	9.5E-01	2.9E-01	NA	NA	NA	NA	NA	NA	NA	NA	NA
Energy metabolism	4.9E-01	5.1E-01	9.4E-01	2.3E-01	9.5E-01	NA	NA	NA	NA	NA	NA	NA	NA
Genetic information processing	**1.1E-04**	**7.7E-05**	1.6E-01	5.1E-01	2.3E-01	2.3E-01	NA	NA	NA	NA	NA	NA	NA
Lipid metabolism	8.7E-01	8.7E-01	4.9E-01	5.4E-01	5.1E-01	4.6E-01	9.5E-01	NA	NA	NA	NA	NA	NA
Metabolism	4.8E-01	5.1E-01	9.1E-01	2.3E-01	8.8E-01	8.4E-01	**3.6E-03**	5.1E-01	NA	NA	NA	NA	NA
Metabolism of cofactors and vitamins	6.8E-01	7.0E-01	9.5E-01	3.0E-01	9.3E-01	8.8E-01	2.4E-01	5.5E-01	9.5E-01	NA	NA	NA	NA
Metabolism of other amino acids	9.3E-01	9.3E-01	9.5E-01	4.6E-01	9.3E-01	8.8E-01	5.4E-01	8.4E-01	9.9E-01	9.5E-01	NA	NA	NA
Nucleotide metabolism	8.8E-01	8.8E-01	9.5E-01	4.4E-01	9.3E-01	9.3E-01	5.1E-01	7.0E-01	9.5E-01	9.7E-01	9.5E-01	NA	NA
Organelle targeting genes	2.3E-01	2.4E-01	9.5E-01	2.3E-01	9.3E-01	8.8E-01	**7.7E-05**	4.9E-01	8.8E-01	9.5E-01	9.5E-01	9.5E-01	NA
Signaling and cellular processes	9.1E-01	8.8E-01	4.9E-01	4.6E-01	5.1E-01	4.9E-01	2.3E-01	8.8E-01	4.6E-01	6.2E-01	8.8E-01	8.8E-01	2.3E-01

Table S6

JBM10

	All genes	Unannotated genes	Amino acid metabolism	Biosynthesis of other secondary metabolites	Carbohydrate metabolism	Energy metabolism	Genetic information processing	Lipid metabolism	Metabolism	Metabolism of cofactors and vitamins	Metabolism of other amino acids	Nucleotide metabolism	Organelle targeting genes
Unannotated genes	**8.9E-04**	NA	NA	NA	NA	NA	NA	NA	NA	NA	NA	NA	NA
Amino acid metabolism	3.8E-01	1.7E-01	NA	NA	NA	NA	NA	NA	NA	NA	NA	NA	NA
Biosynthesis of other secondary metabolites	6.2E-01	4.6E-01	8.7E-01	NA	NA	NA	NA	NA	NA	NA	NA	NA	NA
Carbohydrate metabolism	8.7E-01	6.2E-01	7.3E-01	7.3E-01	NA	NA	NA	NA	NA	NA	NA	NA	NA
Energy metabolism	4.1E-02	1.1E-01	2.3E-02	1.0E-01	1.1E-01	NA	NA	NA	NA	NA	NA	NA	NA
Genetic information processing	**1.3E-14**	**9.8E-22**	3.3E-01	7.8E-01	2.2E-01	**5.5E-05**	NA	NA	NA	NA	NA	NA	NA
Lipid metabolism	7.3E-01	9.9E-01	3.1E-01	3.3E-01	6.2E-01	2.9E-01	7.5E-02	NA	NA	NA	NA	NA	NA
Metabolism	7.0E-01	2.1E-01	6.5E-01	7.2E-01	9.9E-01	3.0E-02	**3.1E-03**	5.4E-01	NA	NA	NA	NA	NA
Metabolism of cofactors and vitamins	4.9E-01	2.2E-01	8.9E-01	8.1E-01	8.1E-01	3.0E-02	2.7E-01	4.0E-01	7.3E-01	NA	NA	NA	NA
Metabolism of other amino acids	4.6E-01	6.2E-01	2.9E-01	3.2E-01	4.6E-01	8.0E-01	1.0E-01	6.5E-01	4.0E-01	2.9E-01	NA	NA	NA
Nucleotide metabolism	5.2E-02	3.0E-02	1.1E-01	2.5E-01	1.1E-01	**2.1E-03**	2.5E-01	3.0E-02	7.6E-02	1.0E-01	6.4E-02	NA	NA
Organelle targeting genes	2.3E-01	9.2E-01	1.6E-01	3.8E-01	5.5E-01	1.6E-01	**1.7E-08**	9.7E-01	2.2E-01	2.2E-01	6.3E-01	2.3E-02	NA
Signaling and cellular processes	5.7E-03	**4.6E-05**	8.0E-01	9.9E-01	5.5E-01	**2.1E-03**	1.7E-01	2.2E-01	2.2E-01	7.2E-01	2.2E-01	1.6E-01	**2.1E-03**

Table S7

JBNZ41

	All genes	Unannotated genes	Amino acid metabolism	Biosynthesis of other secondary metabolites	Carbohydrate metabolism	Energy metabolism	Genetic information processing	Lipid metabolism	Metabolism	Metabolism of cofactors and vitamins	Metabolism of other amino acids	Nucleotide metabolism	Organelle targeting genes
Unannotated genes	**3.2E-03**	NA	NA	NA	NA	NA	NA	NA	NA	NA	NA	NA	NA
Amino acid metabolism	3.5E-02	9.5E-03	NA	NA	NA	NA	NA	NA	NA	NA	NA	NA	NA
Biosynthesis of other secondary metabolites	4.8E-01	3.4E-01	8.1E-01	NA	NA	NA	NA	NA	NA	NA	NA	NA	NA
Carbohydrate metabolism	**9.0E-06**	**1.2E-06**	5.5E-02	1.1E-01	NA	NA	NA	NA	NA	NA	NA	NA	NA
Energy metabolism	**4.2E-04**	**1.0E-04**	1.6E-01	2.2E-01	7.0E-01	NA	NA	NA	NA	NA	NA	NA	NA
Genetic information processing	1.0E-02	3.8E-01	**4.0E-03**	2.6E-01	**2.9E-07**	**2.0E-05**	NA	NA	NA	NA	NA	NA	NA
Lipid metabolism	6.8E-01	4.9E-01	6.3E-01	8.2E-01	2.5E-02	8.3E-02	3.4E-01	NA	NA	NA	NA	NA	NA
Metabolism	1.0E-02	**5.3E-04**	5.5E-01	9.4E-01	**3.1E-03**	2.2E-02	**1.5E-04**	8.4E-01	NA	NA	NA	NA	NA
Metabolism of cofactors and vitamins	1.2E-02	**3.4E-03**	7.3E-01	6.3E-01	1.3E-01	3.4E-01	**1.2E-03**	4.1E-01	3.0E-01	NA	NA	NA	NA
Metabolism of other amino acids	2.8E-01	2.0E-01	9.3E-01	7.6E-01	2.6E-01	4.2E-01	1.4E-01	6.3E-01	6.8E-01	9.3E-01	NA	NA	NA
Nucleotide metabolism	1.0E-02	**4.9E-03**	2.6E-01	2.4E-01	8.7E-01	9.3E-01	**3.1E-03**	1.4E-01	9.7E-02	4.1E-01	4.1E-01	NA	NA
Organelle targeting genes	2.5E-01	3.0E-02	2.2E-01	6.9E-01	**1.5E-04**	**3.4E-03**	6.5E-03	9.3E-01	3.6E-01	9.7E-02	4.1E-01	2.6E-02	NA
Signaling and cellular processes	**4.8E-08**	**6.7E-11**	9.1E-01	7.3E-01	1.9E-02	1.1E-01	**8.4E-11**	4.9E-01	2.1E-01	7.6E-01	9.4E-01	2.5E-01	1.0E-02

Table S8

Table S9, S10 and S11: Pairwise Wilcoxon signed-rank tests on gene variation within functional groups between two strains validate differences. P-values (< 0.01) were bold.

JBC07 - JBM10

	All genes	Unannotated genes	Amino acid metabolism	Biosynthesis of other secondary metabolites	Carbohydrate metabolism	Energy metabolism	Genetic information processing	Lipid metabolism	Metabolism	Metabolism of cofactors and vitamins	Metabolism of other amino acids	Nucleotide metabolism	Organelle targeting genes
Unannotated genes	**2.0E-29**	NA	NA	NA	NA	NA	NA	NA	NA	NA	NA	NA	NA
Amino acid metabolism	**8.0E-06**	**4.4E-11**	NA	NA	NA	NA	NA	NA	NA	NA	NA	NA	NA
Biosynthesis of other secondary metabolites	3.2E-02	**2.1E-03**	6.8E-01	NA	NA	NA	NA	NA	NA	NA	NA	NA	NA
Carbohydrate metabolism	8.3E-01	1.4E-01	3.4E-02	7.4E-02	NA	NA	NA	NA	NA	NA	NA	NA	NA
Energy metabolism	3.6E-01	3.6E-02	1.3E-01	9.8E-02	6.8E-01	NA	NA	NA	NA	NA	NA	NA	NA
Genetic information processing	**1.4E-34**	**2.2E-75**	4.2E-01	5.1E-01	4.7E-02	2.1E-01	NA	NA	NA	NA	NA	NA	NA
Lipid metabolism	5.6E-01	1.2E-01	9.8E-02	1.3E-01	8.3E-01	8.9E-01	1.8E-01	NA	NA	NA	NA	NA	NA
Metabolism	**6.5E-14**	**1.3E-30**	4.3E-01	5.3E-01	3.9E-02	2.1E-01	8.3E-01	1.8E-01	NA	NA	NA	NA	NA
Metabolism of cofactors and vitamins	2.0E-01	**2.2E-03**	7.1E-02	1.3E-01	5.6E-01	1.0E+00	1.3E-01	8.3E-01	1.3E-01	NA	NA	NA	NA
Metabolism of other amino acids	1.4E-02	**6.1E-04**	7.1E-01	8.3E-01	7.1E-02	1.6E-01	4.5E-01	1.3E-01	5.0E-01	1.3E-01	NA	NA	NA
Nucleotide metabolism	**8.0E-06**	**3.9E-08**	3.8E-02	2.8E-01	**8.0E-04**	**2.1E-03**	1.4E-02	**2.1E-03**	1.1E-02	**1.0E-03**	2.4E-01	NA	NA
Organelle targeting genes	2.6E-01	1.8E-02	1.4E-01	2.8E-01	5.1E-01	8.9E-01	2.8E-01	7.8E-01	2.3E-01	8.9E-01	2.2E-01	6.7E-03	NA
Signaling and cellular processes	**8.2E-11**	**6.7E-29**	1.3E-01	2.6E-01	1.4E-01	4.4E-01	1.7E-01	3.6E-01	2.2E-01	3.4E-01	2.3E-01	**2.6E-03**	5.6E-01

Table S9

JBC07 - JBNZ41

	All genes	Unannotated genes	Amino acid metabolism	Biosynthesis of other secondary metabolites	Carbohydrate metabolism	Energy metabolism	Genetic information processing	Lipid metabolism	Metabolism	Metabolism of cofactors and vitamins	Metabolism of other amino acids	Nucleotide metabolism	Organelle targeting genes
Unannotated genes	**5.0E-08**	NA	NA	NA	NA	NA	NA	NA	NA	NA	NA	NA	NA
Amino acid metabolism	7.6E-02	**3.4E-03**	NA	NA	NA	NA	NA	NA	NA	NA	NA	NA	NA
Biosynthesis of other secondary metabolites	9.6E-01	9.1E-01	4.4E-01	NA	NA	NA	NA	NA	NA	NA	NA	NA	NA
Carbohydrate metabolism	4.2E-01	1.6E-01	9.6E-01	6.8E-01	NA	NA	NA	NA	NA	NA	NA	NA	NA
Energy metabolism	4.2E-01	1.5E-01	9.2E-01	6.8E-01	9.7E-01	NA	NA	NA	NA	NA	NA	NA	NA
Genetic information processing	**4.9E-16**	**1.3E-28**	9.2E-01	4.2E-01	9.1E-01	8.7E-01	NA	NA	NA	NA	NA	NA	NA
Lipid metabolism	8.5E-01	4.6E-01	6.8E-01	8.7E-01	8.5E-01	9.2E-01	5.8E-01	NA	NA	NA	NA	NA	NA
Metabolism	1.7E-02	**5.3E-06**	6.8E-01	6.8E-01	9.2E-01	9.6E-01	1.6E-01	9.1E-01	NA	NA	NA	NA	NA
Metabolism of cofactors and vitamins	3.5E-01	7.5E-02	9.1E-01	6.8E-01	9.6E-01	9.6E-01	6.9E-01	9.1E-01	9.4E-01	NA	NA	NA	NA
Metabolism of other amino acids	9.6E-02	3.0E-02	6.0E-01	3.0E-01	6.0E-01	6.0E-01	6.0E-01	3.9E-01	3.5E-01	4.6E-01	NA	NA	NA
Nucleotide metabolism	4.7E-01	2.4E-01	9.7E-01	6.7E-01	9.6E-01	9.2E-01	9.6E-01	6.8E-01	9.1E-01	9.4E-01	7.6E-01	NA	NA
Organelle targeting genes	2.5E-01	9.6E-01	8.9E-03	9.1E-01	1.6E-01	1.5E-01	**9.4E-07**	4.2E-01	**3.4E-03**	9.6E-02	3.2E-02	2.1E-01	NA
Signaling and cellular processes	**3.4E-03**	**7.9E-08**	7.6E-01	6.8E-01	9.4E-01	9.5E-01	1.6E-01	9.1E-01	9.6E-01	9.6E-01	3.5E-01	9.1E-01	**3.4E-03**

Table S10

JBM10 - JBNZ41

	All genes	Unannotated genes	Amino acid metabolism	Biosynthesis of other secondary metabolites	Carbohydrate metabolism	Energy metabolism	Genetic information processing	Lipid metabolism	Metabolism	Metabolism of cofactors and vitamins	Metabolism of other amino acids	Nucleotide metabolism	Organelle targeting genes
Unannotated genes	**2.7E-16**	NA	NA	NA	NA	NA	NA	NA	NA	NA	NA	NA	NA
Amino acid metabolism	**2.3E-03**	**1.7E-06**	NA	NA	NA	NA	NA	NA	NA	NA	NA	NA	NA
Biosynthesis of other secondary metabolites	5.5E-01	2.0E-01	7.8E-01	NA	NA	NA	NA	NA	NA	NA	NA	NA	NA
Carbohydrate metabolism	1.2E-01	7.3E-03	8.7E-01	8.4E-01	NA	NA	NA	NA	NA	NA	NA	NA	NA
Energy metabolism	7.8E-01	2.4E-01	2.4E-01	6.6E-01	4.4E-01	NA	NA	NA	NA	NA	NA	NA	NA
Genetic information processing	**1.9E-19**	**2.3E-41**	7.9E-01	8.4E-01	9.9E-01	2.4E-01	NA	NA	NA	NA	NA	NA	NA
Lipid metabolism	3.1E-01	5.3E-02	8.4E-01	9.9E-01	8.5E-01	6.3E-01	8.5E-01	NA	NA	NA	NA	NA	NA
Metabolism	**3.2E-06**	**2.1E-14**	6.8E-01	8.9E-01	9.0E-01	3.6E-01	7.9E-01	9.8E-01	NA	NA	NA	NA	NA
Metabolism of cofactors and vitamins	5.6E-01	5.0E-02	2.4E-01	7.7E-01	5.3E-01	9.0E-01	2.4E-01	6.3E-01	3.9E-01	NA	NA	NA	NA
Metabolism of other amino acids	1.4E-01	2.6E-02	8.5E-01	6.6E-01	7.2E-01	3.6E-01	6.8E-01	6.8E-01	6.8E-01	3.6E-01	NA	NA	NA
Nucleotide metabolism	8.8E-02	1.4E-02	7.8E-01	6.5E-01	7.0E-01	2.4E-01	6.6E-01	6.6E-01	6.2E-01	2.4E-01	9.9E-01	NA	NA
Organelle targeting genes	9.0E-01	5.3E-02	1.7E-02	6.3E-01	2.0E-01	8.4E-01	**1.0E-03**	3.8E-01	1.4E-02	6.3E-01	2.0E-01	1.3E-01	NA
Signaling and cellular processes	**2.9E-07**	**8.6E-18**	6.2E-01	9.5E-01	8.4E-01	4.1E-01	6.1E-01	9.9E-01	8.7E-01	4.5E-01	6.2E-01	5.7E-01	1.8E-02

Table S11

Table S12: ANOVA test for intraspecific mutation distribution.

P-values < 0.01 are marked bold. The mutation rates of each strain differ in the groups *all genes, unannotated genes* an *signaling and cellular processes* in the amount of SNPs, inserts and deletions per gene. In the groups *genetic information processing* and *organelle targeted genes* only the proportion of SNPs differ signifcantly.

Metabolic_pathway	SNPs	inserts	deletions
All genes	**1.1E-71**	**2.6E-16**	**2.5E-16**
Unannotated genes	**2.9E-88**	**1.5E-20**	**9.0E-19**
Amino acid metabolism	8.5E-01	9.1E-01	8.1E-01
Biosynthesis of other secondary metabolites	2.0E-01	1.4E-01	6.7E-02
Carbohydrate metabolism	1.7E-02	4.5E-01	1.1E-02
Energy metabolism	1.1E-01	2.5E-01	2.4E-01
Genetic information processing	**4.9E-04**	4.4E-01	1.6E-01
Lipid metabolism	8.4E-02	7.2E-01	7.1E-01
Metabolism	3.0E-01	5.7E-01	3.8E-01
Metabolism of cofactors and vitamins	4.8E-01	6.8E-01	3.0E-01
Metabolism of other amino acids	4.9E-01	9.3E-01	9.2E-01
Nucleotide metabolism	4.6E-02	1.8E-01	1.2E-01
Organelle targeted genes	**2.1E-03**	2.4E-02	6.8E-02
Signaling and cellular processes	**2.6E-04**	**4.9E-04**	**4.1E-04**

Table S13:Kruskal-Wallis test for interspecific mutation distribution.

P-values < 0.01 are marked bold. The mutation rates of each strain are consistent in the groups *Biosynthesis of other secondary metabolites, Metabolism of other amino acids* and *Nucleotide metabolism* in the amount of SNPs, inserts and deletions per gene.

Metabolic_pathway	SNPs	inserts	deletions
All genes	**0.0E+00**	**1.7E-104**	**8.9E-134**
Unannotated genes	**3.6E-256**	**1.8E-61**	**2.2E-73**
Amino acid metabolism	**7.9E-07**	**4.7E-03**	**3.3E-04**
Biosynthesis of other secondary metabolites	4.8E-01	3.1E-01	9.6E-01
Carbohydrate metabolism	**9.1E-06**	9.9E-01	**1.2E-03**
Energy metabolism	**1.1E-04**	1.3E-02	5.5E-02
Genetic information processing	**2.8E-85**	**2.1E-22**	**2.9E-30**
Lipid metabolism	**4.3E-03**	6.6E-02	**1.1E-03**
Metabolism	**5.4E-19**	**1.4E-06**	**2.8E-07**
Metabolism of cofactors and vitamins	**1.7E-07**	1.9E-02	**3.2E-05**
Metabolism of other amino acids	**1.0E-03**	5.6E-02	9.3E-01
Nucleotide metabolism	2.8E-01	2.5E-01	3.1E-01
Organelle targeted genes	**9.2E-04**	8.6E-02	6.5E-02
Signaling and cellular processes	**6.5E-32**	**4.5E-05**	**2.5E-11**

Table S14: The KEGG Mapper - Reconstruct Module confirmed metabolic pathways in which max one gene is missing.

ID	Pathway hierarchy L1	L2	L3	strain		
M00009	Carbohydrate and lipid metabolism	Central carbohydrate metabolism	Citrate cycle (TCA cycle, Krebs cycle)	JBC07	JBM10	JBNZ41
M00010	Carbohydrate and lipid metabolism	Central carbohydrate metabolism	Citrate cycle, first carbon oxidation, oxaloacetate => 2-oxoglutarate	JBC07	JBM10	JBNZ41
M00011	Carbohydrate and lipid metabolism	Central carbohydrate metabolism	Citrate cycle, second carbon oxidation, 2-oxoglutarate => oxaloacetate	JBC07	JBM10	JBNZ41
M00008	Carbohydrate and lipid metabolism	Central carbohydrate metabolism	Entner-Doudoroff pathway, glucose-6P => glyceraldehyde-3P + pyruvate			JBNZ41
M00003	Carbohydrate and lipid metabolism	Central carbohydrate metabolism	Gluconeogenesis, oxaloacetate => fructose-6P	JBC07	JBM10	JBNZ41
M00001	Carbohydrate and lipid metabolism	Central carbohydrate metabolism	Glycolysis (Embden-Meyerhof pathway), glucose => pyruvate	JBC07	JBM10	JBNZ41
M00002	Carbohydrate and lipid metabolism	Central carbohydrate metabolism	Glycolysis, core module involving three-carbon compounds	JBC07	JBM10	JBNZ41
M00004	Carbohydrate and lipid metabolism	Central carbohydrate metabolism	Pentose phosphate pathway (Pentose phosphate cycle)			JBNZ41
M00580	Carbohydrate and lipid metabolism	Central carbohydrate metabolism	Pentose phosphate pathway, archaea, fructose 6P => ribose 5P	JBC07	JBM10	JBNZ41
M00007	Carbohydrate and lipid metabolism	Central carbohydrate metabolism	Pentose phosphate pathway, non-oxidative phase, fructose 6P => ribose 5P	JBC07	JBM10	JBNZ41
M00006	Carbohydrate and lipid metabolism	Central carbohydrate metabolism	Pentose phosphate pathway, oxidative phase, glucose 6P => ribulose 5P			JBNZ41
M00005	Carbohydrate and lipid metabolism	Central carbohydrate metabolism	PRPP biosynthesis, ribose 5P => PRPP	JBC07	JBM10	JBNZ41
M00307	Carbohydrate and lipid metabolism	Central carbohydrate metabolism	Pyruvate oxidation, pyruvate => acetyl-CoA	JBC07	JBM10	JBNZ41
M00308	Carbohydrate and lipid metabolism	Central carbohydrate metabolism	Semi-phosphorylative Entner-Doudoroff pathway, gluconate => glycerate-3P	JBC07		
M00087	Carbohydrate and lipid metabolism	Fatty acid metabolism	beta-Oxidation	JBC07	JBM10	JBNZ41
M00086	Carbohydrate and lipid metabolism	Fatty acid metabolism	beta-Oxidation, acyl-CoA synthesis	JBC07	JBM10	JBNZ41
M00083	Carbohydrate and lipid metabolism	Fatty acid metabolism	Fatty acid biosynthesis, elongation	JBC07	JBM10	JBNZ41
M00415	Carbohydrate and lipid metabolism	Fatty acid metabolism	Fatty acid biosynthesis, elongation, endoplasmic reticulum	JBC07	JBM10	JBNZ41
M00085	Carbohydrate and lipid metabolism	Fatty acid metabolism	Fatty acid biosynthesis, elongation, mitochondria	JBC07	JBM10	JBNZ41
M00082	Carbohydrate and lipid metabolism	Fatty acid metabolism	Fatty acid biosynthesis, initiation	JBC07	JBM10	JBNZ41
M00088	Carbohydrate and lipid metabolism	Fatty acid metabolism	Ketone body biosynthesis, acetyl-CoA => acetoacetate/3-hydroxybutyrate/acetone	JBC07	JBM10	JBNZ41
M00055	Carbohydrate and lipid metabolism	Glycan metabolism	N-glycan precursor biosynthesis	JBC07	JBM10	JBNZ41
M00073	Carbohydrate and lipid metabolism	Glycan metabolism	N-glycan precursor trimming	JBC07	JBM10	JBNZ41
M00072	Carbohydrate and lipid metabolism	Glycan metabolism	N-glycosylation by oligosaccharyltransferase	JBC07	JBM10	JBNZ41
M00098	Carbohydrate and lipid metabolism	Lipid metabolism	Acylglycerol degradation	JBC07	JBM10	JBNZ41
M00094	Carbohydrate and lipid metabolism	Lipid metabolism	Ceramide biosynthesis	JBC07	JBM10	JBNZ41
M00130	Carbohydrate and lipid metabolism	Lipid metabolism	Inositol phosphate metabolism, PI=> PIP2 => Ins(1,4,5)P3 => Ins(1,3,4,5)P4	JBC07	JBM10	JBNZ41
M00090	Carbohydrate and lipid metabolism	Lipid metabolism	Phosphatidylcholine (PC) biosynthesis, choline => PC	JBC07	JBM10	JBNZ41
M00092	Carbohydrate and lipid metabolism	Lipid metabolism	Phosphatidylethanolamine (PE) biosynthesis, ethanolamine => PE	JBC07	JBM10	JBNZ41
M00093	Carbohydrate and lipid metabolism	Lipid metabolism	Phosphatidylethanolamine (PE) biosynthesis, PA => PS => PE			JBNZ41
M00099	Carbohydrate and lipid metabolism	Lipid metabolism	Sphingosine biosynthesis	JBC07	JBM10	JBNZ41
M00100	Carbohydrate and lipid metabolism	Lipid metabolism	Sphingosine degradation	JBC07	JBM10	JBNZ41
M00089	Carbohydrate and lipid metabolism	Lipid metabolism	Triacylglycerol biosynthesis	JBC07	JBM10	JBNZ41
M00064	Carbohydrate and lipid metabolism	Lipopolysaccharide metabolism	ADP-L-glycero-D-manno-heptose biosynthesis			JBNZ41
M00063	Carbohydrate and lipid metabolism	Lipopolysaccharide metabolism	CMP-KDO biosynthesis			JBNZ41
M00793	Carbohydrate and lipid metabolism	Other carbohydrate metabolism	dTDP-L-rhamnose biosynthesis			JBNZ41
M00632	Carbohydrate and lipid metabolism	Other carbohydrate metabolism	Galactose degradation, Leloir pathway, galactose => alpha-D-glucose-1P	JBC07	JBM10	JBNZ41
M00012	Carbohydrate and lipid metabolism	Other carbohydrate metabolism	Glyoxylate cycle	JBC07	JBM10	JBNZ41
M00013	Carbohydrate and lipid metabolism	Other carbohydrate metabolism	Malonate semialdehyde pathway, propanoyl-CoA => acetyl-CoA	JBC07	JBM10	JBNZ41
M00554	Carbohydrate and lipid metabolism	Other carbohydrate metabolism	Nucleotide sugar biosynthesis, galactose => UDP-galactose			JBNZ41
M00549	Carbohydrate and lipid metabolism	Other carbohydrate metabolism	Nucleotide sugar biosynthesis, glucose => UDP-glucose	JBC07	JBM10	JBNZ41
M00741	Carbohydrate and lipid metabolism	Other carbohydrate metabolism	Propanoyl-CoA metabolism, propanoyl-CoA => succinyl-CoA	JBC07	JBM10	JBNZ41
M00097	Carbohydrate and lipid metabolism	Other terpenoid biosynthesis	beta-Carotene biosynthesis, GGAP => beta-carotene	JBC07	JBM10	JBNZ41
M00364	Carbohydrate and lipid metabolism	Terpenoid backbone biosynthesis	C10-C20 isoprenoid biosynthesis, bacteria	JBC07	JBM10	JBNZ41
M00367	Carbohydrate and lipid metabolism	Terpenoid backbone biosynthesis	C10-C20 isoprenoid biosynthesis, non-plant eukaryotes	JBC07	JBM10	JBNZ41
M00366	Carbohydrate and lipid metabolism	Terpenoid backbone biosynthesis	C10-C20 isoprenoid biosynthesis, plants	JBC07	JBM10	JBNZ41
M00095	Carbohydrate and lipid metabolism	Terpenoid backbone biosynthesis	C5 isoprenoid biosynthesis, mevalonate pathway	JBC07	JBM10	JBNZ41
M00695	Cellular processes	Cell signaling	cAMP signaling	JBC07	JBM10	JBNZ41
M00692	Cellular processes	Cell signaling	Cell cycle - G1/S transition	JBC07	JBM10	JBNZ41
M00693	Cellular processes	Cell signaling	Cell cycle - G2/M transition	JBC07	JBM10	JBNZ41
M00694	Cellular processes	Cell signaling	cGMP signaling	JBC07	JBM10	JBNZ41
M00691	Cellular processes	Cell signaling	DNA damage-induced cell cycle checkpoints	JBC07	JBM10	JBNZ41
M00687	Cellular processes	Cell signaling	MAPK (ERK1/2) signaling	JBC07	JBM10	JBNZ41
M00689	Cellular processes	Cell signaling	MAPK (p38) signaling	JBC07	JBM10	JBNZ41
M00152	Energy metabolism	ATP synthesis	Cytochrome bc1 complex	JBC07	JBM10	JBNZ41
M00151	Energy metabolism	ATP synthesis	Cytochrome bc1 complex respiratory unit	JBC07	JBM10	JBNZ41
M00153	Energy metabolism	ATP synthesis	Cytochrome bd ubiquinol oxidase		JBM10	
M00154	Energy metabolism	ATP synthesis	Cytochrome c oxidase	JBC07	JBM10	JBNZ41
M00156	Energy metabolism	ATP synthesis	Cytochrome c oxidase, cbb3-type	JBC07	JBM10	JBNZ41
M00155	Energy metabolism	ATP synthesis	Cytochrome c oxidase, prokaryotes	JBC07		JBNZ41
M00158	Energy metabolism	ATP synthesis	F-type ATPase, eukaryotes	JBC07	JBM10	JBNZ41
M00157	Energy metabolism	ATP synthesis	F-type ATPase, prokaryotes and chloroplasts	JBC07	JBM10	JBNZ41
M00145	Energy metabolism	ATP synthesis	NAD(P)H:quinone oxidoreductase, chloroplasts and cyanobacteria	JBC07		JBNZ41
M00146	Energy metabolism	ATP synthesis	NADH dehydrogenase (ubiquinone) 1 alpha subcomplex	JBC07	JBM10	JBNZ41
M00147	Energy metabolism	ATP synthesis	NADH dehydrogenase (ubiquinone) 1 beta subcomplex	JBC07	JBM10	JBNZ41

M00143	Energy metabolism	ATP synthesis	NADH dehydrogenase (ubiquinone) Fe-S protein/flavoprotein complex, mitochondria	JBC07	JBM10	JBNZ41
M00144	Energy metabolism	ATP synthesis	NADH:quinone oxidoreductase, prokaryotes	JBC07	JBM10	JBNZ41
M00148	Energy metabolism	ATP synthesis	Succinate dehydrogenase (ubiquinone)	JBC07	JBM10	JBNZ41
M00149	Energy metabolism	ATP synthesis	Succinate dehydrogenase, prokaryotes	JBC07		JBNZ41
M00160	Energy metabolism	ATP synthesis	V-type ATPase, eukaryotes	JBC07	JBM10	JBNZ41
M00171	Energy metabolism	Carbon fixation	C4-dicarboxylic acid cycle, NAD - malic enzyme type	JBC07	JBM10	JBNZ41
M00172	Energy metabolism	Carbon fixation	C4-dicarboxylic acid cycle, NADP - malic enzyme type	JBC07	JBM10	JBNZ41
M00170	Energy metabolism	Carbon fixation	C4-dicarboxylic acid cycle, phosphoenolpyruvate carboxykinase type	JBC07	JBM10	JBNZ41
M00168	Energy metabolism	Carbon fixation	CAM (Crassulacean acid metabolism), dark	JBC07	JBM10	JBNZ41
M00169	Energy metabolism	Carbon fixation	CAM (Crassulacean acid metabolism), light	JBC07	JBM10	JBNZ41
M00579	Energy metabolism	Carbon fixation	Phosphate acetyltransferase-acetate kinase pathway, acetyl-CoA => acetate	JBC07	JBM10	JBNZ41
M00165	Energy metabolism	Carbon fixation	Reductive pentose phosphate cycle (Calvin cycle)	JBC07	JBM10	JBNZ41
M00167	Energy metabolism	Carbon fixation	Reductive pentose phosphate cycle, glyceraldehyde-3P => ribulose-5P	JBC07	JBM10	JBNZ41
M00166	Energy metabolism	Carbon fixation	Reductive pentose phosphate cycle, ribulose-5P => glyceraldehyde-3P	JBC07	JBM10	JBNZ41
M00597	Energy metabolism	Photosynthesis	Anoxygenic photosystem II		JBM10	JBNZ41
M00161	Energy metabolism	Photosynthesis	Photosystem II	JBC07	JBM10	JBNZ41
M00176	Energy metabolism	Sulfur metabolism	Assimilatory sulfate reduction, sulfate => H2S	JBC07	JBM10	JBNZ41
M00254	Environmental information processing	ABC-2 type and other transport systems	ABC-2 type transport system	JBC07	JBM10	JBNZ41
M00256	Environmental information processing	ABC-2 type and other transport systems	Cell division transport system			JBNZ41
M00259	Environmental information processing	ABC-2 type and other transport systems	Heme transport system			JBNZ41
M00252	Environmental information processing	ABC-2 type and other transport systems	Lipooligosaccharide transport system	JBC07		JBNZ41
M00320	Environmental information processing	ABC-2 type and other transport systems	Lipopolysaccharide export system	JBC07		JBNZ41
M00250	Environmental information processing	ABC-2 type and other transport systems	Lipopolysaccharide transport system		JBM10	JBNZ41
M00255	Environmental information processing	ABC-2 type and other transport systems	Lipoprotein-releasing system		JBM10	JBNZ41
M00258	Environmental information processing	ABC-2 type and other transport systems	Putative ABC transport system	JBC07	JBM10	JBNZ41
M00330	Environmental information processing	Bacterial secretion system	Adhesin protein transport system	JBC07	JBM10	JBNZ41
M00571	Environmental information processing	Bacterial secretion system	AlgE-type Mannuronan C-5-Epimerase transport system	JBC07	JBM10	JBNZ41
M00325	Environmental information processing	Bacterial secretion system	alpha-Hemolysin/cyclolysin transport system	JBC07	JBM10	JBNZ41
M00429	Environmental information processing	Bacterial secretion system	Competence-related DNA transformation transporter	JBC07	JBM10	JBNZ41
M00339	Environmental information processing	Bacterial secretion system	RaxAB-RaxC type I secretion system	JBC07	JBM10	JBNZ41
M00326	Environmental information processing	Bacterial secretion system	RTX toxin transport system	JBC07	JBM10	JBNZ41
M00335	Environmental information processing	Bacterial secretion system	Sec (secretion) system	JBC07	JBM10	JBNZ41
M00336	Environmental information processing	Bacterial secretion system	Twin-arginine translocation (Tat) system			JBNZ41
M00331	Environmental information processing	Bacterial secretion system	Type II general secretion system	JBC07	JBM10	JBNZ41
M00333	Environmental information processing	Bacterial secretion system	Type IV secretion system	JBC07	JBM10	JBNZ41
M00334	Environmental information processing	Bacterial secretion system	Type VI secretion system			JBNZ41
M00646	Environmental information processing	Drug efflux transporter/pump	Multidrug resistance, efflux pump AcrAD-TolC			JBNZ41
M00648	Environmental information processing	Drug efflux transporter/pump	Multidrug resistance, efflux pump MdtABC			JBNZ41
M00717	Environmental information processing	Drug efflux transporter/pump	Multidrug resistance, efflux pump NorA	JBC07	JBM10	JBNZ41
M00821	Environmental information processing	Drug efflux transporter/pump	Multidrug resistance, efflux pump TriABC-TolC	JBC07	JBM10	JBNZ41
M00720	Environmental information processing	Drug efflux transporter/pump	Multidrug resistance, efflux pump VexEF-TolC	JBC07	JBM10	JBNZ41
M00742	Environmental information processing	Drug resistance	Aminoglycoside resistance, protease FtsH	JBC07	JBM10	JBNZ41
M00743	Environmental information processing	Drug resistance	Aminoglycoside resistance, protease HtpX			JBNZ41
M00729	Environmental information processing	Drug resistance	Fluoroquinolone resistance, gyrase-protecting protein Qnr	JBC07		
M00247	Environmental information processing	Metallic cation, iron-siderophore and vitamin B12 transport system	Putative ABC transport system			JBNZ41
M00208	Environmental information processing	Mineral and organic ion transport system	Glycine betaine/proline transport system			JBNZ41
M00190	Environmental information processing	Mineral and organic ion transport system	Iron(III) transport system			JBNZ41
M00189	Environmental information processing	Mineral and organic ion transport system	Molybdate transport system	JBC07		JBNZ41
M00188	Environmental information processing	Mineral and organic ion transport system	NitT/TauT family transport system		JBM10	JBNZ41
M00193	Environmental information processing	Mineral and organic ion transport system	Putative spermidine/putrescine transport system	JBC07	JBM10	JBNZ41
M00192	Environmental information processing	Mineral and organic ion transport system	Putative thiamine transport system	JBC07	JBM10	JBNZ41
M00299	Environmental information processing	Mineral and organic ion transport system	Spermidine/putrescine transport system	JBM10		JBNZ41
M00185	Environmental information processing	Mineral and organic ion transport system	Sulfate transport system	JBC07		JBNZ41
M00436	Environmental information processing	Mineral and organic ion transport system	Sulfonate transport system	JBM10		JBNZ41
M00349	Environmental information processing	Peptide and nickel transport system	Microcin C transport system			JBNZ41
M00239	Environmental information processing	Peptide and nickel transport system	Peptides/nickel transport system	JBC07		JBNZ41
M00237	Environmental information processing	Phosphate and amino acid transport system	Branched-chain amino acid transport system		JBM10	JBNZ41
M00222	Environmental information processing	Phosphate and amino acid transport system	Phosphate transport system			JBNZ41
M00236	Environmental information processing	Phosphate and amino acid transport system	Putative polar amino acid transport system		JBM10	JBNZ41

M00219	Environmental information processing	Saccharide, polyol, and lipid transport system	AI-2 transport system		JBC07	JBNZ41
M00201	Environmental information processing	Saccharide, polyol, and lipid transport system	alpha-Glucoside transport system			JBNZ41
M00491	Environmental information processing	Saccharide, polyol, and lipid transport system	arabinogalactan oligomer/maltooligosaccharide transport system			JBNZ41
M00602	Environmental information processing	Saccharide, polyol, and lipid transport system	Arabinosaccharide transport system			JBNZ41
M00206	Environmental information processing	Saccharide, polyol, and lipid transport system	Cellobiose transport system			JBNZ41
M00669	Environmental information processing	Saccharide, polyol, and lipid transport system	gamma-Hexachlorocyclohexane transport system	JBC07		JBNZ41
M00605	Environmental information processing	Saccharide, polyol, and lipid transport system	Glucose/mannose transport system			JBNZ41
M00194	Environmental information processing	Saccharide, polyol, and lipid transport system	Maltose/maltodextrin transport system			JBNZ41
M00670	Environmental information processing	Saccharide, polyol, and lipid transport system	Mce transport system	JBC07		JBNZ41
M00196	Environmental information processing	Saccharide, polyol, and lipid transport system	Multiple sugar transport system			JBNZ41
M00606	Environmental information processing	Saccharide, polyol, and lipid transport system	N,'N''-Diacetylchitobiose transport system			JBNZ41
M00210	Environmental information processing	Saccharide, polyol, and lipid transport system	Phospholipid transport system	JBC07		JBNZ41
M00211	Environmental information processing	Saccharide, polyol, and lipid transport system	Putative ABC transport system	JBC07	JBM10	JBNZ41
M00197	Environmental information processing	Saccharide, polyol, and lipid transport system	Putative fructooligosaccharide transport system			JBNZ41
M00207	Environmental information processing	Saccharide, polyol, and lipid transport system	Putative multiple sugar transport system			JBNZ41
M00221	Environmental information processing	Saccharide, polyol, and lipid transport system	Putative simple sugar transport system	JBC07	JBM10	JBNZ41
M00200	Environmental information processing	Saccharide, polyol, and lipid transport system	Putative sorbitol/mannitol transport system			JBNZ41
M00475	Environmental information processing	Two-component regulatory system	BarA-UvrY (central carbon metabolism) two-component regulatory system			JBNZ41
M00512	Environmental information processing	Two-component regulatory system	CckA-CtrA/CpdR (cell cycle control) two-component regulatory system	JBC07		JBNZ41
M00506	Environmental information processing	Two-component regulatory system	CheA-CheYBV (chemotaxis) two-component regulatory system			JBNZ41
M00452	Environmental information processing	Two-component regulatory system	CusS-CusR (copper tolerance) two-component regulatory system			JBM10
M00445	Environmental information processing	Two-component regulatory system	EnvZ-OmpR (osmotic stress response) two-component regulatory system			JBNZ41
M00524	Environmental information processing	Two-component regulatory system	FixL-FixJ (nitrogen fixation) two-component regulatory system		JBM10	JBNZ41
M00497	Environmental information processing	Two-component regulatory system	GlnL-GlnG (nitrogen regulation) two-component regulatory system			JBNZ41
M00454	Environmental information processing	Two-component regulatory system	KdpD-KdpE (potassium transport) two-component regulatory system			JBNZ41
M00498	Environmental information processing	Two-component regulatory system	NtrY-NtrX (nitrogen regulation) two-component regulatory system	JBC07		JBNZ41
M00434	Environmental information processing	Two-component regulatory system	PhoR-PhoB (phosphate starvation response) two-component regulatory system			JBNZ41
M00511	Environmental information processing	Two-component regulatory system	PleC-PleD (cell fate control) two-component regulatory system	JBC07	JBM10	JBNZ41
M00523	Environmental information processing	Two-component regulatory system	RegB-RegA (redox response) two-component regulatory system			JBNZ41
M00516	Environmental information processing	Two-component regulatory system	SLN1-YPD1-SSK1/SKN7 (osmosensing) two-component regulatory system	JBC07	JBM10	JBNZ41
M00745	Gene set	Drug resistance	Imipenem resistance, repression of porin OprD		JBM10	
M00261	Genetic information processing	DNA polymerase	DNA polymerase alpha / primase complex	JBC07	JBM10	JBNZ41
M00262	Genetic information processing	DNA polymerase	DNA polymerase delta complex	JBC07	JBM10	JBNZ41
M00263	Genetic information processing	DNA polymerase	DNA polymerase epsilon complex	JBC07	JBM10	JBNZ41
M00260	Genetic information processing	DNA polymerase	DNA polymerase III complex, bacteria	JBC07	JBM10	JBNZ41
M00293	Genetic information processing	DNA polymerase	DNA polymerase zeta complex	JBC07	JBM10	JBNZ41
M00343	Genetic information processing	Proteasome	Archaeal proteasome	JBC07	JBM10	JBNZ41
M00342	Genetic information processing	Proteasome	Bacterial proteasome	JBC07	JBM10	JBNZ41
M00337	Genetic information processing	Proteasome	Immunoproteasome	JBC07	JBM10	JBNZ41
M00341	Genetic information processing	Proteasome	Proteasome, 19S regulatory particle (PA700)	JBC07	JBM10	JBNZ41
M00340	Genetic information processing	Proteasome	Proteasome, 20S core particle	JBC07	JBM10	JBNZ41
M00404	Genetic information processing	Protein processing	COPII complex	JBC07	JBM10	JBNZ41
M00408	Genetic information processing	Protein processing	ESCRT-0 complex	JBC07	JBM10	JBNZ41
M00409	Genetic information processing	Protein processing	ESCRT-I complex	JBC07	JBM10	JBNZ41
M00410	Genetic information processing	Protein processing	ESCRT-II complex	JBC07	JBM10	JBNZ41
M00412	Genetic information processing	Protein processing	ESCRT-III complex	JBC07	JBM10	JBNZ41
M00403	Genetic information processing	Protein processing	HRD1/SEL1 ERAD complex	JBC07	JBM10	JBNZ41
M00400	Genetic information processing	Protein processing	p97-Ufd1-Npl4 complex	JBC07	JBM10	JBNZ41
M00401	Genetic information processing	Protein processing	Sec61 complex	JBC07	JBM10	JBNZ41
M00402	Genetic information processing	Protein processing	Translocon-associated protein (TRAP) complex	JBC07	JBM10	JBNZ41
M00296	Genetic information processing	Repair system	BER complex	JBC07	JBM10	JBNZ41
'M00414	Genetic information processing	Repair system	Bloom''s syndrome complex	JBC07	JBM10	JBNZ41'
M00295	Genetic information processing	Repair system	BRCA1-associated genome surveillance complex (BASC)	JBC07	JBM10	JBNZ41
M00297	Genetic information processing	Repair system	DNA-PK complex	JBC07	JBM10	JBNZ41
M00413	Genetic information processing	Repair system	FA core complex	JBC07	JBM10	JBNZ41
M00290	Genetic information processing	Repair system	Holo-TFIIH complex	JBC07	JBM10	JBNZ41

M00291	Genetic information processing	Repair system	MRN complex	JBC07	JBM10	JBNZ41
M00292	Genetic information processing	Repair system	MRX complex	JBC07	JBM10	JBNZ41
M00286	Genetic information processing	Replication system	GINS complex	JBC07	JBM10	JBNZ41
M00285	Genetic information processing	Replication system	MCM complex	JBC07	JBM10	JBNZ41
M00284	Genetic information processing	Replication system	Origin recognition complex	JBC07	JBM10	JBNZ41
M00289	Genetic information processing	Replication system	RF-C complex	JBC07	JBM10	JBNZ41
M00288	Genetic information processing	Replication system	RPA complex	JBC07	JBM10	JBNZ41
M00179	Genetic information processing	Ribosome	Ribosome, archaea	JBC07	JBM10	JBNZ41
M00178	Genetic information processing	Ribosome	Ribosome, bacteria	JBC07	JBM10	JBNZ41
M00177	Genetic information processing	Ribosome	Ribosome, eukaryotes	JBC07	JBM10	JBNZ41
M00182	Genetic information processing	RNA polymerase	RNA polymerase I, eukaryotes	JBC07	JBM10	JBNZ41
M00180	Genetic information processing	RNA polymerase	RNA polymerase II, eukaryotes	JBC07	JBM10	JBNZ41
M00181	Genetic information processing	RNA polymerase	RNA polymerase III, eukaryotes	JBC07	JBM10	JBNZ41
M00183	Genetic information processing	RNA polymerase	RNA polymerase, bacteria	JBC07	JBM10	JBNZ41
M00395	Genetic information processing	RNA processing	Decapping complex	JBC07	JBM10	JBNZ41
M00428	Genetic information processing	RNA processing	eIF4F complex	JBC07	JBM10	JBNZ41
M00430	Genetic information processing	RNA processing	Exon junction complex (EJC)	JBC07	JBM10	JBNZ41
M00390	Genetic information processing	RNA processing	Exosome, archaea	JBC07	JBM10	JBNZ41
M00391	Genetic information processing	RNA processing	Exosome, eukaryotes	JBC07	JBM10	JBNZ41
M00425	Genetic information processing	RNA processing	H/ACA ribonucleoprotein complex	JBC07	JBM10	JBNZ41
M00427	Genetic information processing	RNA processing	Nuclear pore complex	JBC07	JBM10	JBNZ41
M00394	Genetic information processing	RNA processing	RNA degradosome	JBC07	JBM10	JBNZ41
M00392	Genetic information processing	RNA processing	Ski complex	JBC07	JBM10	JBNZ41
M00426	Genetic information processing	RNA processing	Survival motor neuron (SMN) complex	JBC07	JBM10	JBNZ41
M00405	Genetic information processing	RNA processing	THC complex	JBC07	JBM10	JBNZ41
M00393	Genetic information processing	RNA processing	TRAMP complex	JBC07	JBM10	JBNZ41
M00406	Genetic information processing	RNA processing	TREX complex	JBC07	JBM10	JBNZ41
M00399	Genetic information processing	Spliceosome	Cap binding complex	JBC07	JBM10	JBNZ41
M00397	Genetic information processing	Spliceosome	Lsm 1-7 complex	JBC07	JBM10	JBNZ41
M00396	Genetic information processing	Spliceosome	Lsm 2-8 complex	JBC07	JBM10	JBNZ41
M00398	Genetic information processing	Spliceosome	Sm core complex	JBC07	JBM10	JBNZ41
M00355	Genetic information processing	Spliceosome	Spliceosome, 35S U5-snRNP	JBC07	JBM10	JBNZ41
M00353	Genetic information processing	Spliceosome	Spliceosome, Prp19/CDC5L complex	JBC07	JBM10	JBNZ41
M00351	Genetic information processing	Spliceosome	Spliceosome, U1-snRNP	JBC07	JBM10	JBNZ41
M00352	Genetic information processing	Spliceosome	Spliceosome, U2-snRNP	JBC07	JBM10	JBNZ41
M00354	Genetic information processing	Spliceosome	Spliceosome, U4/U6.U5 tri-snRNP	JBC07	JBM10	JBNZ41
M00389	Genetic information processing	Ubiquitin system	APC/C complex	JBC07	JBM10	JBNZ41
M00384	Genetic information processing	Ubiquitin system	Cul3-SPOP complex	JBC07	JBM10	JBNZ41
M00386	Genetic information processing	Ubiquitin system	Cul4-DDB1-CSA complex	JBC07	JBM10	JBNZ41
M00385	Genetic information processing	Ubiquitin system	Cul4-DDB1-DDB2 complex	JBC07	JBM10	JBNZ41
M00388	Genetic information processing	Ubiquitin system	ECS complex	JBC07	JBM10	JBNZ41
M00383	Genetic information processing	Ubiquitin system	ECV complex	JBC07	JBM10	JBNZ41
M00380	Genetic information processing	Ubiquitin system	SCF-BTRC complex	JBC07	JBM10	JBNZ41
M00407	Genetic information processing	Ubiquitin system	SCF-CDC4 complex	JBC07	JBM10	JBNZ41
M00382	Genetic information processing	Ubiquitin system	SCF-FBS complex	JBC07	JBM10	JBNZ41
M00387	Genetic information processing	Ubiquitin system	SCF-FBW7 complex	JBC07	JBM10	JBNZ41
M00411	Genetic information processing	Ubiquitin system	SCF-GRR1 complex	JBC07	JBM10	JBNZ41
M00379	Genetic information processing	Ubiquitin system	SCF-MET30 complex	JBC07	JBM10	JBNZ41
M00381	Genetic information processing	Ubiquitin system	SCF-SKP2 complex	JBC07	JBM10	JBNZ41
M00359	Metabolism	Aminoacyl tRNA	Aminoacyl-tRNA biosynthesis, eukaryotes	JBC07	JBM10	JBNZ41
M00360	Metabolism	Aminoacyl tRNA	Aminoacyl-tRNA biosynthesis, prokaryotes	JBC07	JBM10	JBNZ41
M00361	Metabolism	Nucleotide sugar	Nucleotide sugar biosynthesis, eukaryotes	JBC07	JBM10	JBNZ41
M00362	Metabolism	Nucleotide sugar	Nucleotide sugar biosynthesis, prokaryotes			JBNZ41
M00844	Nucleotide and amino acid metabolism	Arginine and proline metabolism	Arginine biosynthesis, ornithine => arginine	JBC07	JBM10	JBNZ41
M00028	Nucleotide and amino acid metabolism	Arginine and proline metabolism	Ornithine biosynthesis, glutamate => ornithine	JBC07	JBM10	JBNZ41
M00015	Nucleotide and amino acid metabolism	Arginine and proline metabolism	Proline biosynthesis, glutamate => proline	JBC07	JBM10	JBNZ41
M00029	Nucleotide and amino acid metabolism	Arginine and proline metabolism	Urea cycle		JBM10	JBNZ41
M00024	Nucleotide and amino acid metabolism	Aromatic amino acid metabolism	Phenylalanine biosynthesis, chorismate => phenylalanine	JBC07	JBM10	JBNZ41
M00022	Nucleotide and amino acid metabolism	Aromatic amino acid metabolism	Shikimate pathway, phosphoenolpyruvate + erythrose-4P => chorismate	JBC07	JBM10	JBNZ41
M00038	Nucleotide and amino acid metabolism	Aromatic amino acid metabolism	Tryptophan metabolism, tryptophan => kynurenine => 2-aminomuconate			JBC07
M00025	Nucleotide and amino acid metabolism	Aromatic amino acid metabolism	Tyrosine biosynthesis, chorismate => tyrosine	JBC07	JBM10	JBNZ41
M00040	Nucleotide and amino acid metabolism	Aromatic amino acid metabolism	Tyrosine biosynthesis, prephanate => pretyrosine => tyrosine			JBNZ41
M00044	Nucleotide and amino acid metabolism	Aromatic amino acid metabolism	Tyrosine degradation, tyrosine => homogentisate		JBM10	JBNZ41
M00535	Nucleotide and amino acid metabolism	Branched-chain amino acid metabolism	Isoleucine biosynthesis, pyruvate => 2-oxobutanoate			JBNZ41
M00570	Nucleotide and amino acid metabolism	Branched-chain amino acid metabolism	Isoleucine biosynthesis, threonine => 2-oxobutanoate => isoleucine			JBNZ41
M00432	Nucleotide and amino acid metabolism	Branched-chain amino acid metabolism	Leucine biosynthesis, 2-oxoisovalerate => 2-oxoisocaproate			JBNZ41
M00036	Nucleotide and amino acid metabolism	Branched-chain amino acid metabolism	Leucine degradation, leucine => acetoacetate + acetyl-CoA	JBC07	JBM10	JBNZ41
M00019	Nucleotide and amino acid metabolism	Branched-chain amino acid metabolism	Valine/isoleucine biosynthesis, pyruvate => valine / 2-oxobutanoate => isoleucine			JBNZ41
M00141	Nucleotide and amino acid metabolism	Cofactor and vitamin biosynthesis	C1-unit interconversion, eukaryotes	JBC07	JBM10	JBNZ41
M00140	Nucleotide and amino acid metabolism	Cofactor and vitamin biosynthesis	C1-unit interconversion, prokaryotes	JBC07	JBM10	JBNZ41
M00120	Nucleotide and amino acid metabolism	Cofactor and vitamin biosynthesis	Coenzyme A biosynthesis, pantothenate => CoA	JBC07	JBM10	JBNZ41
M00121	Nucleotide and amino acid metabolism	Cofactor and vitamin biosynthesis	Heme biosynthesis, glutamate => heme	JBC07	JBM10	JBNZ41
M00843	Nucleotide and amino acid metabolism	Cofactor and vitamin biosynthesis	L-threo-Tetrahydrobiopterin biosynthesis, GTP => L-threo-BH4	JBC07	JBM10	JBNZ41
M00115	Nucleotide and amino acid metabolism	Cofactor and vitamin biosynthesis	NAD biosynthesis, aspartate => NAD	JBC07		JBNZ41
M00119	Nucleotide and amino acid metabolism	Cofactor and vitamin biosynthesis	Pantothenate biosynthesis, valine/L-aspartate => pantothenate	JBC07		JBNZ41

M00572	Nucleotide and amino acid metabolism	Cofactor and vitamin biosynthesis	Pimeloyl-ACP biosynthesis, BioC-BioH pathway, malonyl-ACP => pimeloyl-ACP			JBNZ41
M00125	Nucleotide and amino acid metabolism	Cofactor and vitamin biosynthesis	Riboflavin biosynthesis, GTP => riboflavin/FMN/FAD	JBC07	JBM10	JBNZ41
M00846	Nucleotide and amino acid metabolism	Cofactor and vitamin biosynthesis	Siroheme biosynthesis, glutamate => siroheme			JBNZ41
M00842	Nucleotide and amino acid metabolism	Cofactor and vitamin biosynthesis	Tetrahydrobiopterin biosynthesis, GTP => BH4	JBC07	JBM10	JBNZ41
M00126	Nucleotide and amino acid metabolism	Cofactor and vitamin biosynthesis	Tetrahydrofolate biosynthesis, GTP => THF	JBC07	JBM10	JBNZ41
M00841	Nucleotide and amino acid metabolism	Cofactor and vitamin biosynthesis	Tetrahydrofolate biosynthesis, mediated by PTPS, GTP => THF	JBC07		
M00112	Nucleotide and amino acid metabolism	Cofactor and vitamin biosynthesis	Tocopherol/tocotorienol biosynthesis	JBC07	JBM10	JBNZ41
M00021	Nucleotide and amino acid metabolism	Cysteine and methionine metabolism	Cysteine biosynthesis, serine => cysteine	JBC07	JBM10	JBNZ41
M00017	Nucleotide and amino acid metabolism	Cysteine and methionine metabolism	Methionine biosynthesis, apartate => homoserine => methionine			JBNZ41
M00034	Nucleotide and amino acid metabolism	Cysteine and methionine metabolism	Methionine salvage pathway	JBC07	JBM10	JBNZ41
M00026	Nucleotide and amino acid metabolism	Histidine metabolism	Histidine biosynthesis, PRPP => histidine	JBC07	JBM10	JBNZ41
M00032	Nucleotide and amino acid metabolism	Lysine metabolism	Lysine degradation, lysine => saccharopine => acetoacetyl-CoA	JBC07	JBM10	JBNZ41
M00134	Nucleotide and amino acid metabolism	Polyamine biosynthesis	Polyamine biosynthesis, arginine => ornithine => putrescine	JBC07	JBM10	JBNZ41
M00049	Nucleotide and amino acid metabolism	Purine metabolism	Adenine ribonucleotide biosynthesis, IMP => ADP,ATP	JBC07	JBM10	JBNZ41
M00050	Nucleotide and amino acid metabolism	Purine metabolism	Guanine ribonucleotide biosynthesis IMP => GDP,GTP	JBC07	JBM10	JBNZ41
M00048	Nucleotide and amino acid metabolism	Purine metabolism	Inosine monophosphate biosynthesis, PRPP + glutamine => IMP	JBC07	JBM10	JBNZ41
M00546	Nucleotide and amino acid metabolism	Purine metabolism	Purine degradation, xanthine => urea	JBC07	JBM10	JBNZ41
M00052	Nucleotide and amino acid metabolism	Pyrimidine metabolism	Pyrimidine ribonucleotide biosynthesis, UMP => UDP/UTP,CDP/CTP	JBC07	JBM10	JBNZ41
M00051	Nucleotide and amino acid metabolism	Pyrimidine metabolism	Uridine monophosphate biosynthesis, glutamine (+ PRPP) => UMP	JBC07	JBM10	JBNZ41
M00555	Nucleotide and amino acid metabolism	Serine and threonine metabolism	Betaine biosynthesis, choline => betaine	JBC07	JBM10	JBNZ41
M00020	Nucleotide and amino acid metabolism	Serine and threonine metabolism	Serine biosynthesis, glycerate-3P => serine	JBC07	JBM10	JBNZ41
M00018	Nucleotide and amino acid metabolism	Serine and threonine metabolism	Threonine biosynthesis, aspartate => homoserine => threonine	JBC07	JBM10	JBNZ41
